# The genetic causal relationship between type 2 diabetes, glycemic traits and venous thromboembolism, deep vein thrombosis, pulmonary embolism: a two-sample Mendelian randomization study

**DOI:** 10.1186/s12959-024-00600-z

**Published:** 2024-03-29

**Authors:** Mingyi Yang, Xianjie Wan, Yani Su, Ke Xu, Pengfei Wen, Binfei Zhang, Lin Liu, Zhi Yang, Peng Xu

**Affiliations:** Department of Joint Surgery, HongHui Hospital, Xi’an Jiaotong University, Xi’an, Shaanxi 710054 China

**Keywords:** Type 2 diabetes, Glycemic traits, Venous thromboembolism, Deep vein thrombosis, Pulmonary embolism

## Abstract

**Objective:**

To investigate the genetic underpinnings of the association between type 2 diabetes (T2D), glycemic indicators such as fasting glucose (FG), fasting insulin (FI), and glycated hemoglobin (GH), and venous thromboembolism (VTE), encompassing deep vein thrombosis (DVT) and pulmonary embolism (PE), thereby contributing novel insights to the scholarly discourse within this domain.

**Methods:**

Genome-wide association study (GWAS) summary data pertaining to exposures (T2D, FG, FI, GH) and outcomes (VTE, DVT, PE) were acquired from the IEU Open GWAS database, encompassing participants of European descent, including both male and female individuals. Two-sample Mendelian randomization (MR) analyses were conducted utilizing the TwoSampleMR and MRPRESSO packages within the R programming environment. The primary analytical approach employed was the random-effects inverse variance weighted (IVW) method. Heterogeneity was assessed via Cochran’s Q statistic for MR-IVW and Rucker’s Q statistic for MR-Egger. Horizontal pleiotropy was evaluated using the intercept test of MR Egger and MR pleiotropy residual sum and outlier (MR-PRESSO) analysis, with the latter also employed for outlier detection. Additionally, a “Leave one out” analysis was conducted to ascertain the influence of individual single nucleotide polymorphisms (SNPs) on MR results.

**Results:**

The random-effects IVW analysis revealed a negative genetic causal association between T2D) and VTE (*P* = 0.008, Odds Ratio [OR] 95% confidence interval [CI] = 0.896 [0.827–0.972]), as well as between FG and VTE (*P* = 0.002, OR 95% CI = 0.655 [0.503–0.853]), GH and VTE (*P* = 0.010, OR 95% CI = 0.604 [0.412–0.884]), and GH and DVT (*P* = 0.002, OR 95% CI = 0.413 [0.235–0.725]). Conversely, the random-effects IVW analysis did not detect a genetic causal relationship between FI and VTE (*P* > 0.05), nor between T2D, FG, or FI and DVT (*P* > 0.05), or between T2D, FG, FI, or GH and PE (*P* > 0.05). Both the Cochran’s Q statistic for MR-IVW and Rucker’s Q statistic for MR-Egger indicated no significant heterogeneity (*P* > 0.05). Moreover, the intercept tests of MR Egger and MR-PRESSO suggested the absence of horizontal pleiotropy (*P* > 0.05). MR-PRESSO analysis identified no outliers, while the “Leave one out” analysis underscored that the MR analysis was not influenced by any single SNP.

**Conclusion:**

Our investigation revealed that T2D, FG, and GH exhibit negative genetic causal relationships with VTE at the genetic level, while GH demonstrates a negative genetic causal relationship with DVT at the genetic level. These findings furnish genetic-level evidence warranting further examination of VTE, DVT, and PE, thereby making a contribution to the advancement of related research domains.

**Supplementary Information:**

The online version contains supplementary material available at 10.1186/s12959-024-00600-z.

## Introduction

Venous thromboembolism (VTE) is a group of diseases that includes deep vein thrombosis (DVT) and pulmonary embolism (PE) [1]. DVT usually occurs in the deep veins of the legs, arms and visceral veins and can be associated with purpura [2]. PE is a disease characterized by the occlusion of one or more pulmonary arteries by embolism [3]. Although embolism caused by anything obstructing the pulmonary artery (thrombus, tumor, fat or air) can be considered a PE, in clinical practice it is most commonly formed by a circulating thrombus entering the pulmonary artery, and most PEs originate in the lower extremity DVT [4]. According to statistics, nearly 10 million people worldwide are affected by VTE every year, and in addition, the annual incidence of DVT is nearly 500,000 cases and the annual incidence of PE is nearly 300,000 cases in 6 European countries with a population of nearly 300 million [2]. The pathogenesis of VTE, DVT and PE is complex, but the main view at this stage is that the three elements of Virchow thrombosis and hereditary or acquired factors. the three elements of Virchow thrombosis are slow blood flow, hypercoagulability and vessel wall damage [5]. Genetic factors play an important role in the development of thromboembolic diseases. The five typical genetic thrombogenic factors are antithrombin deficiency, protein C deficiency, protein S deficiency, resistance to activated protein C caused by factor V Leiden (rs6025) and prothrombin mutation (prothrombin G20210A, rs1799963) [6]. Anticoagulation is currently the first line of treatment for patients with confirmed VTE [7]. In addition, thrombolytic therapy and lower extremity venous filter implantation are also important treatments for patients with VTE, DVT and PE [8]. VTE, DVT and PE are common complications and causes of death in hospitalized patients, with high incidence, high morbidity and mortality rates and high hospitalization costs [2]. VTE, DVT and PE are one of the common complications in orthopedic patients, such as patients after total hip arthroplasty and femoral neck fracture, which are common in orthopedic surgery, due to surgery and activity restriction, resulting in an increased risk of postoperative complications such as VTE, DVT and PE, which need to be given sufficient attention in clinical practice [9–12]. Although current treatments are effective in reducing the morbidity and mortality of VTE, DVT, and PE, the efficacy of VTE, DVT, and PE is not always satisfactory due to their complexity, and more studies are needed to investigate their occurrence and development.

Type 2 diabetes (T2D) is a series of pathophysiological changes caused by long-term elevated blood glucose and the formation of advanced glycation end products (AGEs), which damage vascular endothelial cells and activate platelets and clotting factors. Over the years, numerous studies have found that T2D is associated with a variety of diseases. A large number of studies have found that patients with T2D can develop a significant hypercoagulable state, often combined with microangiopathy (diabetic nephropathy and retinopathy), macrovascular disease (coronary artery disease, stroke) and embolic disease [13]. Biochemical indicators related to human blood glucose levels have been considered as a measure of the severity of diabetes and the risk of related diseases, and elevated fasting glucose (FG) is an important marker of all pre-diabetes and diabetes. Studies have found that patients with abnormal FG values are at significantly higher risk of developing diabetes and cardiovascular disease [14, 15]. Another study found an association between FG variability and the risk of depression [16]. Insulin is a peptide hormone secreted by pancreatic islet cells. Research have found that insulin is the only hormone in the body that lowers blood sugar while promoting glycogen, fat, and protein synthesis. Fasting insulin (FI) is also closely associated with thrombosis [17]. Glycated hemoglobin (GH) levels represent the average level of glycemic control over a 2–3 months period. GH is strongly associated with both diabetic microangiopathy and macroangiopathy, and even GH levels within the normal range are associated with an increased risk of diabetic macrovascular complications [18]. Studies have found that hyperglycemia can also increase the incidence of embolic events by activating platelets and accelerating the production of clotting factors, putting patients in a hypercoagulable state [19]. It has also been suggested that overly strict glycemic control may lead to an increase in adverse events, including embolic events [20]. T2D and glycemic traits are strongly associated with many human diseases, and in many studies, diabetes and glycemic traits have been considered as risk factors for a range of embolic events such as VTE, DVT and PE [21–23]. However, previous studies have not investigated whether diabetes and glycemic traits are causally related to a series of embolic events such as VTE, DVT and PE at the genetic level.

Mendelian Randomization (MR) methods model and infer causal effects through genetic variation. In traditional epidemiological studies, confounding factors can interfere with the causal inference of exposure and outcome, and MR methods using single nucleotide polymorphism (SNP) as instrumental variables (IVs) can theoretically avoid the effects of confounding factors effectively. Currently, MR method was widely used in studies to assess the relationship between exposure factors and diseases [24–26]. In a MR study of blood cells and risk of VTE, genetic susceptibility to high erythrocyte distribution width, mean reticulocyte volume, mean erythrocyte volume, and low monocyte count was found to be associated with a higher risk of VTE [27]. MR methods were applied to study the genetic causality between obesity and VTE [28]. Another MR study found that elevated fibrinogen levels at the genetic level were associated with the risk of PE but not with DVT [29]. One MR study found a causal relationship between increased whole-body iron status and increased risk of T2D [30]. More, the genetic susceptibility of T2D and higher GH levels was found to be associated with a higher risk of large and small vessel ischemic stroke by MR methods [31]. Numerous studies have demonstrated the feasibility and credibility of the MR methods in studying the causal relationship between exposure and outcome at the genetic level. In this study, we research the causal relationship between T2D, glycemic traits (FG, FI, GH) and VTE, DVT, PE at the genetic level using MR methods.

## Materials and methods

### Study design

This study evaluated genetic causality between exposure (T2D, FG, FI, GH) and outcome (VTE, DVT, PE) by MR analysis using IVs, based on the large-scale genome-wide association study (GWAS) summary data. MR analysis must satisfy three assumptions: (1) The IVs selected were strongly associated with exposure; (2) The IVs selected was not associated with any confounding factors; (3) The IVs selected can affect outcomes only through exposure. All GWAS summary data used in this study are publicly available. Ethical permission and written informed consent had been provided in the initial studies. Details of the data are shown in Supplementary Table 1.

### GWAS summary data for exposure (T2D, FG, FI, GH)

GWAS summary data pertaining to exposure variables (T2D, FG, FI, GH) were sourced from the IEU Open GWAS database (https://gwas.mrcieu.ac.uk/). The T2D dataset encompassed 70,127 samples and comprised 14,277,791 SNPs. Notably, all participants in the study were of European descent and represented both male and female individuals. It is pertinent to note that the genotyping data and subject inclusion adhered to the guidelines delineated by the Wellcome Trust Case Control Consortium (WTCCC) [32], whose exclusion criteria align with established quality filters for GWAS [33]. Genotyping was conducted utilizing Affymetrix v6.0 and Illumina 1.2 M chips. Subsequent to variant quality filtering and exclusion of variants with a minor allele frequency (MAF) below 0.01, a two-step genotype imputation methodology was implemented. This involved prephasing the study genotypes into full haplotypes with SHAPEIT2 [34] to mitigate computational demands associated with genotype imputation through IMPUTE2 [35]. For genotype imputation, two sequence-based reference panels were employed: the 1000 Genomes (1000G) Phase 1 release and the UK10K dataset. Comprehensive information regarding the dataset can be found in previously published studies [36].

The GWAS summary data for FG comprised 200,622 samples and 31,008,728 SNPs, whereas FI data encompassed 151,013 samples with 29,664,438 SNPs, and GH data comprised 146,806 samples with 30,649,064 SNPs. All participants hailed from European ancestry, encompassing both male and female individuals. Each participating cohort rigorously conducted study-level quality control (QC), imputation, and association analyses in accordance with a standardized analysis protocol. Genotyping of cohorts was executed using commercially available genome-wide arrays or the Illumina CardioMetabochip (Metabochip) array [37]. Pre-imputation procedures entailed meticulous sample and variant QC to retain solely high-quality variants in the genotype scaffold for imputation. Sample QC protocols involved the exclusion of samples with call rates below 95%, extreme heterozygosity, sex discrepancies with X chromosome variants, duplicate entries, first- or second-degree relatives (unless by design), or individuals deemed as ancestry outliers. Comprehensive information regarding the dataset is available in published studies [38].

### GWAS summary data for outcome (VTE, DVT, PE)

GWAS summary data pertaining to VTE, as well as DVT and PE, were procured from the IEU Open GWAS database, a repository managed by the FinnGen consortium. The dataset for VTE comprised 218,792 individuals (9,176 cases and 209,616 controls), encompassing 16,380,466 SNPs. Similarly, the DVT dataset consisted of 194,604 participants (4,576 cases and 190,028 controls) with 16,380,409 SNPs, while the PE dataset encompassed 218,413 individuals (4,185 cases and 214,228 controls) with 16,380,466 SNPs. Notably, all subjects were of European descent, comprising both male and female individuals. The FinnGen initiative, a collaborative endeavor between public and private entities, amalgamates genetic data concerning disease endpoints from the Finnish Biobank and the Finnish Health Registry [39]. This research endeavor endeavors to discern genotype-phenotype associations within the Finnish populace. Disease cases were defined utilizing the M13 code within the International Classification of Diseases, Tenth Revision (ICD-10). Genotyping was executed utilizing Illumina and Affymetrix chip arrays, collectively analyzing 16,962,023 genetic variants. Further elaboration on the dataset particulars can be obtained from the FinnGen project’s official documentation available on their website.

### IVs selection

In order to ensure the robustness and reliability of MR analysis outcomes, a rigorous series of QC procedures was undertaken for the selection of IVs. Initially, SNPs associated with the exposure (*P* < 5 × 10^− 8^) were identified [40, 41]. Subsequently, recognizing the potential for biased results stemming from strong linkage disequilibrium(LD) among selected SNPs, a clumping procedure was implemented (r^2^ < 0.001, clumping distance = 10,000 kb) [39]. SNPs associated with the outcome (*P* < 5 × 10^− 8^) were subsequently excluded. Moreover, leveraging the PhenoScanner database, efforts were made to identify and exclude potential confounding factors [42]. The principal risk factors for the outcome of interest —namely, chronic immobility, malignant neoplasms, major surgical procedures, obesity, and smoking—were determined through comprehensive literature review [43, 44]. Furthermore, in order to ensure robust association with the exposure, SNPs with F-statistic values exceeding 10 were selected as IVs. F-statistic values were computed utilizing the formula: F = R^2^(N-K-1)/K(1-R^2^), where R^2^ was derived from: R^2^ = 2×MAF×(1-MAF) Beta^2^. Additionally, palindromic SNPs with intermediate allele frequencies were excluded to ensure that the effect of SNPs on exposure corresponded to the same allele exerting an effect on the outcome [45].

### Statistical analysis

Utilizing the TwoSampleMR and MRPRESSO packages within the R statistical environment (version 4.1.2), a two-sample MR analysis was conducted to investigate the associations between exposures and outcomes. The random-effects inverse variance weighted (IVW) method served as the primary analytical approach, supplemented by weighted median and weighted mode methods. Our MR analysis primarily relied on the random-effects IVW method. This approach employs a meta-analytical framework to amalgamate Wald ratio estimates of causal effects derived from various SNPs, furnishing a consistent estimation of the causal relationship between the exposure and outcome, assuming each genetic variant meets the instrumental variable assumptions [46]. Notably, IVW yields reliable estimates when at least half of the instrumental variables utilized are valid [47]. It synthesizes causal estimates from individual SNPs akin to a two-stage least squares or allele score analysis with individual-level data, thereby representing a conventional alternative to MR [48]. The weighted median method furnishes valid estimates when at least 50% of the information originates from valid SNPs [49]. The Weighted mode is sensitive to the difficult bandwidth selection for mode estimation [50].

The Cochran’s Q statistic for MR-IVW and Rucker’s Q statistic for MR Egger were employed to assess the heterogeneity in MR analysis between exposure and outcome variables, with a threshold of *P* > 0.05 indicating absence of heterogeneity [51]. The intercept test of MR Egger and MR pleiotropy residual sum and outlier (MR-PRESSO) were utilized to identify horizontal pleiotropy, where a *P* > 0.05 indicated absence of such pleiotropy [42]. Subsequently, MR-PRESSO analysis was conducted to detect outliers within the MR analysis [52]. Notably, any outliers detected in the MR-PRESSO analysis were excluded, followed by a second round of causal estimation. Furthermore, a “Leave one out” analysis was undertaken to explore whether the causal relationship between exposure and outcome was influenced by individual SNPs [46]. Importantly, to mitigate the potential for false positives or false negatives, a secondary round of genetic estimation was performed subsequent to the removal of any single SNPs that could impact the MR analysis results. Finally, Maximum likelihood, Penalised weighted median, and IVW (fixed effects) methods were employed as validation techniques to further ascertain potential causal associations between exposure and outcome.

## Results

### IVs selection

Following screening for SNPs associated with exposure and subsequent removal of LD, a total of 16 SNPs were identified as associated with T2D, 66 SNPs with FG, 38 SNPs with FI, and 74 SNPs with GH. Additionally, 16 SNPs were found to be shared between T2D and the outcomes VTE, DVT, and PE, while no SNPs were directly associated with these outcomes. Two potentially confounding SNPs (rs9268835 and rs71304101) were excluded, leaving 14 SNPs utilized as IVs with a corresponding F-statistic exceeding 10. None of these SNPs exhibited palindromic characteristics (Supplementary Table 2). Moreover, 65 SNPs were found to be shared between FG and the aforementioned outcomes, with one SNP (rs507666) directly associated with the outcomes being excluded, along with three confounding SNPs (rs10830963, rs174583, rs507666). Additionally, six SNPs with an F-statistic below 10 (rs39713, rs4760278, rs17270243, rs2657879, rs12898997, rs896854) were excluded. Consequently, 56 SNPs were retained as IVs, with an F-statistic exceeding 10, and three of these exhibited palindromic characteristics (rs10487796, rs2238435, rs2302593) (Supplementary Table 3). Furthermore, 38 SNPs were identified as shared between FI and the outcomes VTE and DVT. One SNP (rs75179845) directly associated with these outcomes was excluded, along with one confounding SNP (rs35000407) and 13 SNPs with an F-statistic below 10. This resulted in 23 SNPs being utilized as IVs with an F-statistic exceeding 10 and no palindromic SNPs observed. Additionally, 38 SNPs were shared between FI and the outcome PE. No SNPs were directly associated with PE, but one confounding SNP (rs35000407) and 13 SNPs with an F-statistic below 10 were excluded. Consequently, 24 SNPs were utilized as IVs with an F-statistic exceeding 10, and no palindromic SNPs were identified (Supplementary Table 4). Finally, 71 SNPs were identified as shared between GH and the outcomes VTE, DVT, and PE. One SNP (rs651007) directly associated with these outcomes was excluded, along with two confounding SNPs (rs10830963, rs651007), and 49 SNPs (including rs651007) with an F-statistic below 10. This left 21 SNPs utilized as IVs with an F-statistic exceeding 10, and no palindromic SNPs were present (Supplementary Table 5).

### MR analysis of exposure (T2D, FG, FI, GH) and VTE

**Fig. 1:**
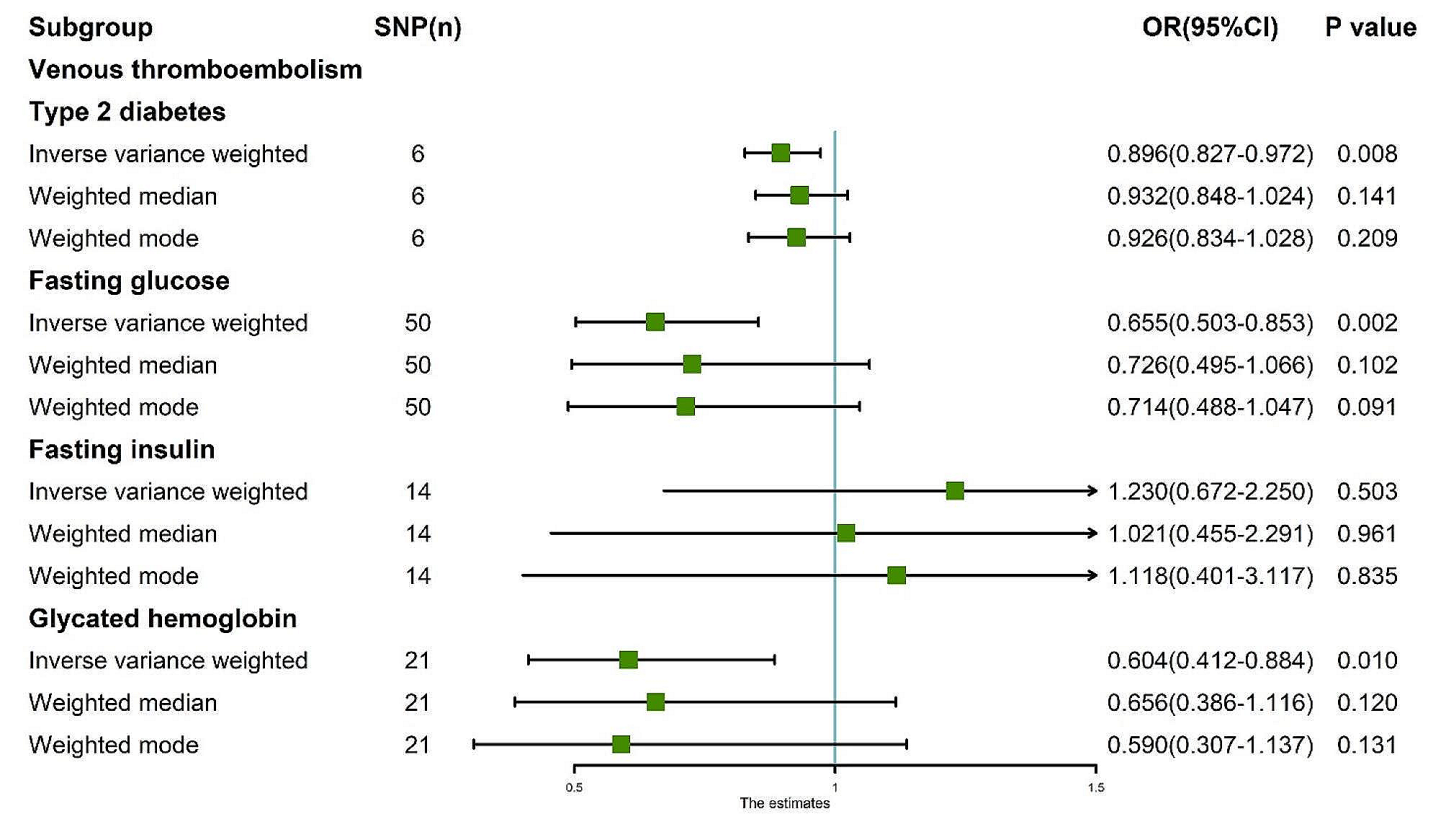
MR analysis results of the exposure (T2D, FG, FI, GH) and VTE. Three methods: random-effects IVW, weighted median and weighted mode

**Fig. 2:**
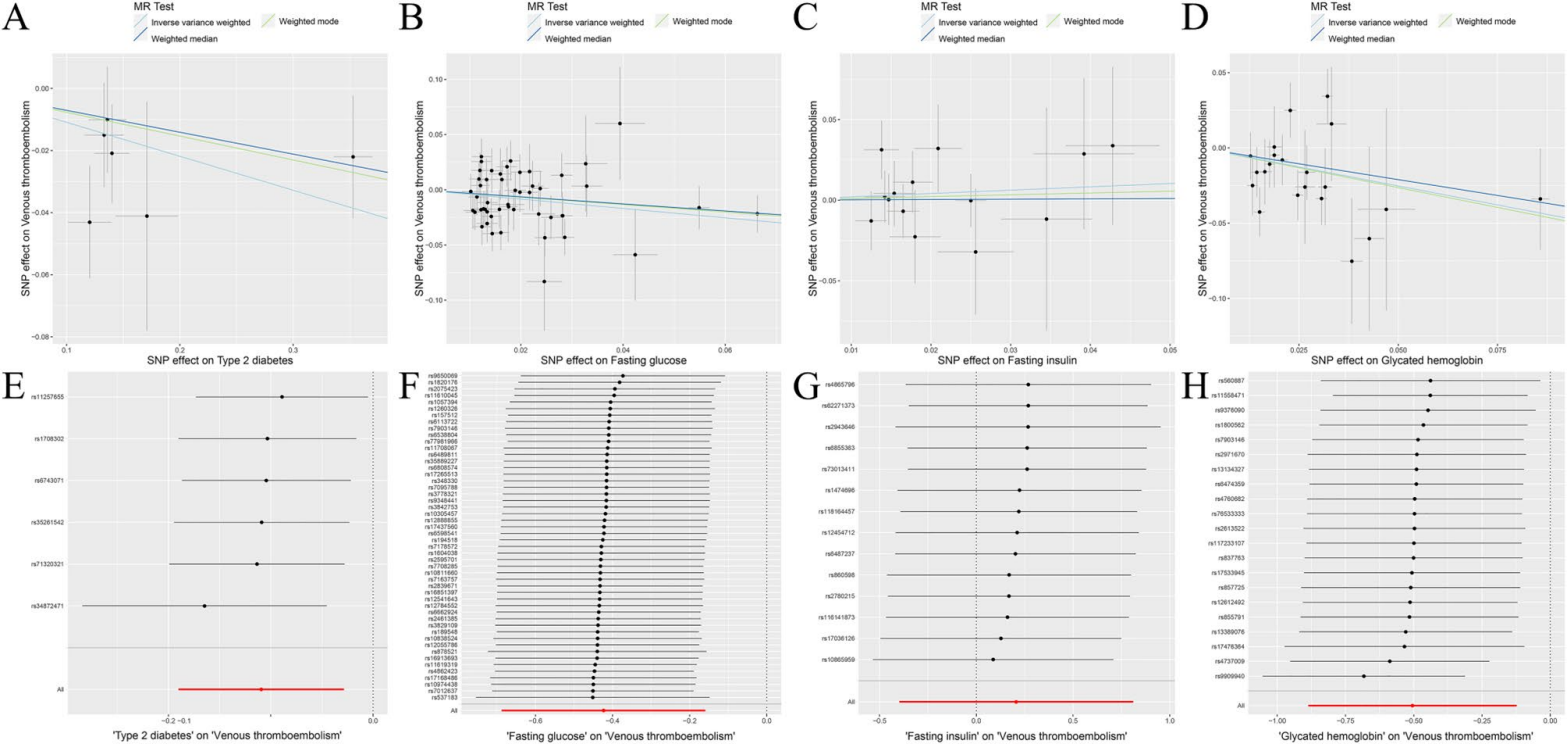
MR analysis results of the exposure (T2D, FG, FI, GH) and VTE. **A**: Scatter plot of T2D and VTE; **B**: Scatter plot of FG and VTE; **C**: Scatter plot of FI and VTE; **D**: Scatter plot of GH and VTE; **E**: Leave one out analysis of T2D and VTE; **F**: Leave one out analysis of FG and VTE; **G**: Leave one out analysis of FI and VTE; **H**: Leave one out analysis of GH and VTE

**Fig. 3:**
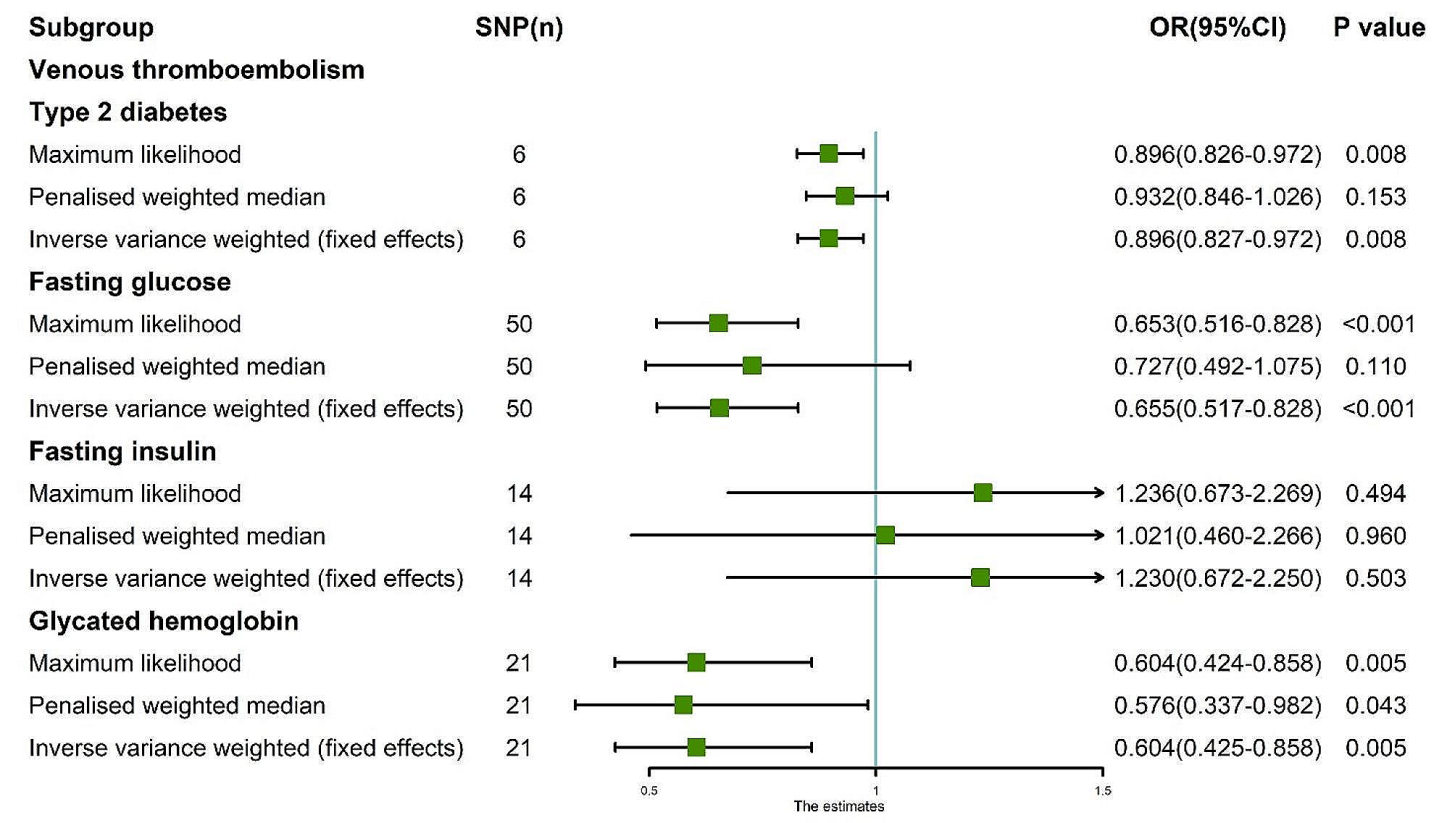
MR analysis results of the exposure (T2D, FG, FI, GH) and VTE. Three methods: maximum likelihood, penalised weighted median, and IVW (fixed effects)

The random-effects IVW results showed that T2D had no genetic causal relationship with VTE (Supplementary Fig. 1A, 1D). Both Cochran’s Q statistic of MR-IVW and Rucker’s Q statistic of MR Egger indicated an absence of heterogeneity (*P* > 0.05). Similarly, the intercept test of MR Egger and MR-PRESSO revealed no evidence of horizontal pleiotropy (*P* > 0.05). Furthermore, the MR-PRESSO analysis detected no outliers (Supplementary Table 6). However, subsequent “Leave-one-out” analysis revealed that the MR analysis was influenced by a single SNP (two SNPs) (Supplementary Fig. 1D). Consequently, a secondary MR analysis was conducted following the removal of these SNPs, which led to a change in the direction of the observed genetic causal relationship, with T2D exhibiting a negative association with VTE (Supplementary Fig. 1B, 1E). This iteration of MR analysis maintain consistency in terms of heterogeneity, absence of horizontal pleiotropy, and outliers (Supplementary Table 6). Despite this, the “Leave-one-out” analysis again identified a SNP (one SNP) driving the results (Supplementary Fig. 1E), prompting a further round of analysis wherein one SNP was removed. Subsequent results from this round reverted to indicating no genetic causal relationship between T2D and VTE, accompanied by consistent findings regarding heterogeneity, horizontal pleiotropy, and outliers (Supplementary Fig. 1C, 1F; Supplementary Table 6). However, the “Leave-one-out” analysis revealed that the results were influenced by five SNPs (Supplementary Fig. 1F), necessitating a fourth round of MR analysis following their exclusion. Notably, this final analysis demonstrated a negative genetic causal relationship between T2D and VTE (*P* = 0.008, Odds Ratio [OR] 95% confidence interval [CI] = 0.896 [0.827–0.972]) as per the random-effects IVW method. Additionally, Weighted Median and Weighted Mode analyses indicated no genetic causal relationship between T2D and VTE (Figs. 1 and 2A). The MR analysis had no heterogeneity and horizontal pleiotropy (*P* > 0.05), and outliers (Table 1). The absence of a single SNP driving the results was confirmed by the “Leave-one-out” analysis (Fig. 2E). Furthermore, the Maximum Likelihood and IVW (fixed effects) analyses corroborated the findings of the random-effects IVW method (*P* < 0.05). Finally, Penalized Weighted Median analysis indicated no genetic causal relationship between T2D and VTE (*P* > 0.05) (Fig. 3).

**Table 1:** Sensitivity analysis of the MR analysis results of exposure and outcome

Exposure	Outcome	Heterogeneity Test		Pleiotropy Test	MR-PRESSO	
		Cochran’s Q Test (IVW)	Rucker’s Q Test (MR-Egger)	Egger Intercept (MR-Egger)	Outliers	Pleiotropy
		*P* value	*P* value	*P* value	Number	*P* value
T2D	VTE	0.548	0.684	0.259	0	0.559
FG		0.103	0.089	0.722	0	0.057
FI		0.896	0.851	0.874	0	0.890
GH		0.260	0.258	0.355	0	0.237
T2D	DVT	0.463	0.394	0.720	0	0.520
FG		0.279	0.246	0.975	0	0.255
FI		0.896	0.875	0.545	0	0.866
GH		0.144	0.120	0.635	0	0.123
T2D	PE	0.940	0.899	0.894	0	0.951
FG		0.027	0.021	0.907	0	0.063
FI		0.633	0.681	0.209	0	0.617
GH		0.678	0.707	0.257	0	0.646

MR: Mendelian randomization; T2D: Type 2 diabetes; FG: Fasting glucose; FI: Fasting insulin; GH: Glycated hemoglobin; VTE: Venous thromboembolism; DVT: Deep vein thrombosis; PE: Pulmonary embolism

The random-effects IVW analysis revealed that there was no genetic causal relationship between FG and VTE (Supplementary Fig. 2A, 2C). Both the Cochran’s Q statistic of MR-IVW and Rucker’s Q statistic of MR Egger indicated significant heterogeneity (*P* < 0.05). The intercept test of MR Egger did not detect horizontal pleiotropy (*P* > 0.05); however, the MR-PRESSO test indicated the presence of horizontal pleiotropy (*P* < 0.05). Further MR-PRESSO analysis identified one outlier (rs10838693) and one potential outlier (rs78132593) (Supplementary Table 6). Additionally, the “Leave one out” analysis suggested that the MR analysis was heavily influenced by a single SNP (11 SNPs) (Supplementary Fig. 2C). Consequently, a second round of MR analysis was conducted after excluding rs10838693. Subsequent random-effects IVW analysis revealed a negative genetic causal relationship between FG and VTE (Supplementary Fig. 2B, 2D). However, heterogeneity persisted in the MR analysis (*P* < 0.05). While the intercept test of MR Egger did not detect horizontal pleiotropy (*P* > 0.05), MR-PRESSO indicated the presence of horizontal pleiotropy (*P* < 0.05), identifying two potential outliers (rs78132593, rs11603349) (Supplementary Table 6). Notably, the “Leave one out” analysis suggested that the MR analysis results were not driven by a single SNP (Supplementary Fig. 2D). Subsequent to excluding these potential outliers, a third round of MR analysis was undertaken. The random-effects IVW analysis revealed a negative genetic causal relationship between FG and VTE (*P* = 0.002, OR 95% CI = 0.655 [0.503–0.853]). Both the Weighted Median and Weighted Mode methods indicated no genetic causal relationship between FG and VTE (Figs. 1 and 2B). Heterogeneity and horizontal pleiotropy were not observed in the MR analysis (*P* > 0.05), nor were there any outliers (Table 1). Moreover, the “Leave one out” analysis suggested that the MR analysis was not influenced by a single SNP (Fig. 2F). Additionally, the Maximum Likelihood and IVW (fixed effects) analyses were consistent with the random-effects IVW findings (*P* < 0.05). Furthermore, Penalised Weighted Median analysis indicated no genetic causal relationship between FG and VTE (*P* > 0.05) (Fig. 3).

The random-effects IVW analysis revealed no genetic causal relationship between FI and VTE (Supplementary Fig. 3A, 3C). Both the Cochran’s Q statistic for MR-IVW and Rucker’s Q statistic for MR Egger suggested an absence of heterogeneity (*P* > 0.05). Furthermore, the intercept tests of MR Egger and MR-PRESSO indicated no evidence of horizontal pleiotropy (*P* > 0.05). Subsequent MR-PRESSO analysis revealed no outliers (Supplementary Table 6). However, “Leave one out” analysis identified that the MR analysis was influenced by a single SNP (one SNP) (Supplementary Fig. 3C). Consequently, we conducted a second round of MR analysis after removing this single SNP. The random-effects IVW analysis then demonstrated a negative genetic causal relationship between FI and VTE (Supplementary Fig. 3B, 3D). This subsequent MR analysis also showed no heterogeneity, horizontal pleiotropy, or outliers (Supplementary Table 6). Nonetheless, “Leave one out” analysis indicated that the MR analysis was influenced by a different set of SNPs, specifically eight SNPs (Supplementary Fig. 3D). Consequently, we carried out a third round of MR analysis, excluding these eight SNPs. The random-effects IVW analysis from this iteration suggested once again that FI had no genetic causal relationship with VTE (*P* = 0.503, OR 95% CI = 1.230 [0.672–2.250]). Results from Weighted Median and Weighted Mode analyses corroborated those of the random-effects IVW (Figs. 1 and 2C). Additionally, this MR analysis exhibited no heterogeneity, horizontal pleiotropy (*P* > 0.05), or outliers (Table 1). Notably, “Leave one out” analysis indicated that the MR analysis was not influenced by a single SNP (Fig. 2G). Moreover, results from Maximum Likelihood, Penalised Weighted Median, and IVW (Fixed Effects) analyses were consistent with those of the random-effects IVW (*P* > 0.05) (Fig. 3).

The random-effects IVW analysis revealed a negative genetic causal association between GH and VTE (*P* = 0.010, OR 95% CI = 0.604 [0.412–0.884]). Contrarily, both the Weighted Median and Weighted Mode estimations suggested an absence of genetic causal relationship between GH and VTE (Figs. 1 and 2D). The assessments of heterogeneity using Cochran’s Q statistic for MR-IVW and Rucker’s Q statistic for MR Egger demonstrated no significant heterogeneity (*P* > 0.05). Furthermore, the intercept tests conducted via MR Egger and MR-PRESSO indicated no evidence of horizontal pleiotropy (*P* > 0.05). Subsequent MR-PRESSO analysis confirmed the absence of outliers (Table 1). Moreover, the “Leave-one-out” analysis suggested that the MR analysis was no influenced by any single SNP (Fig. 2H). Lastly, the consistency of findings was supported by the Maximum Likelihood, Penalised Weighted Median, and IVW (fixed effects) analyses, all of which corroborated the results of the random-effects IVW analysis (*P* < 0.05) (Fig. 3).

### MR analysis of exposure (T2D, FG, FI, GH) and DVT

**Fig. 4:**
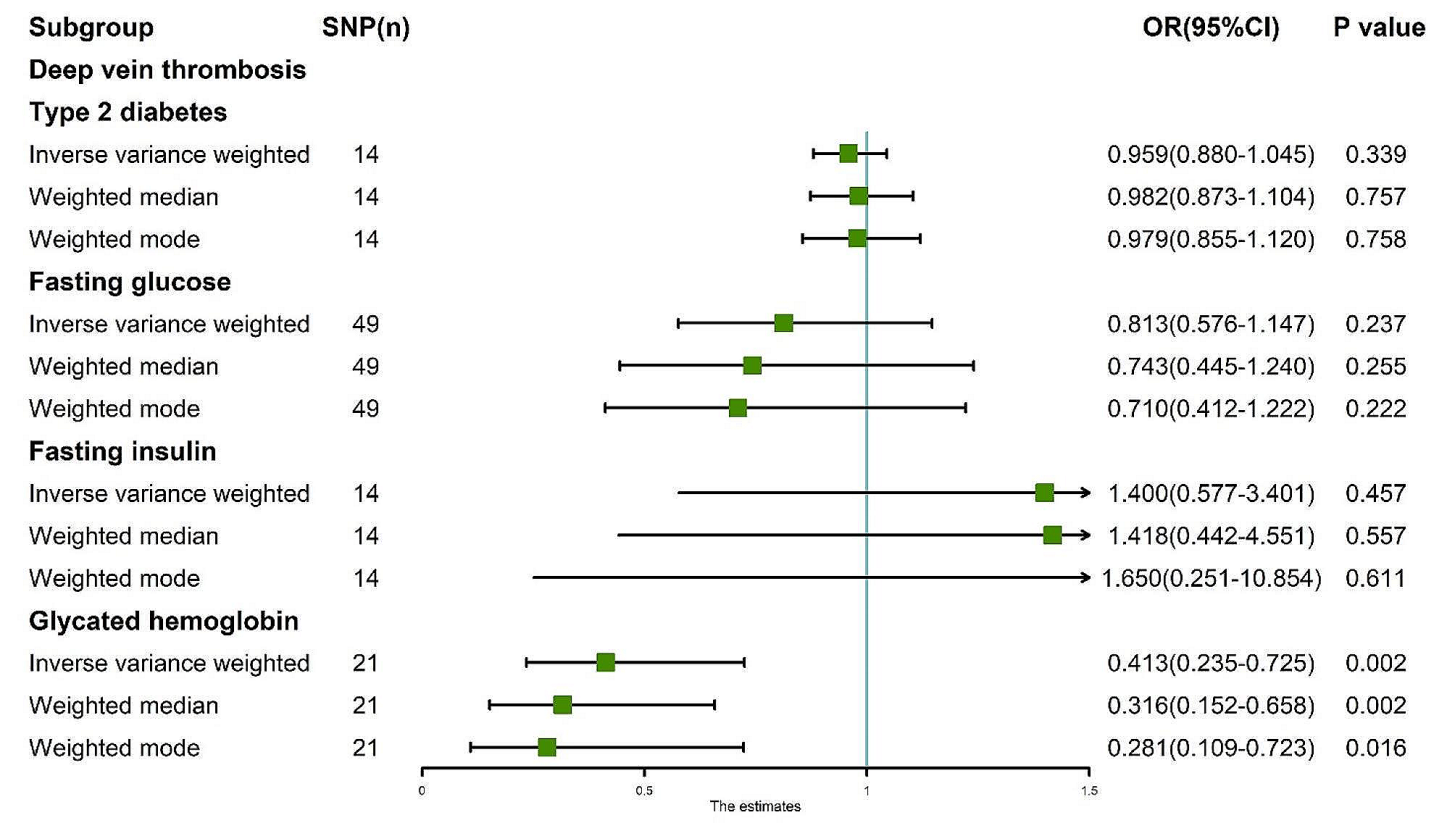
MR analysis results of the exposure (T2D, FG, FI, GH) and DVT. Three methods: random-effects IVW, weighted median and weighted mode

**Fig. 5:**
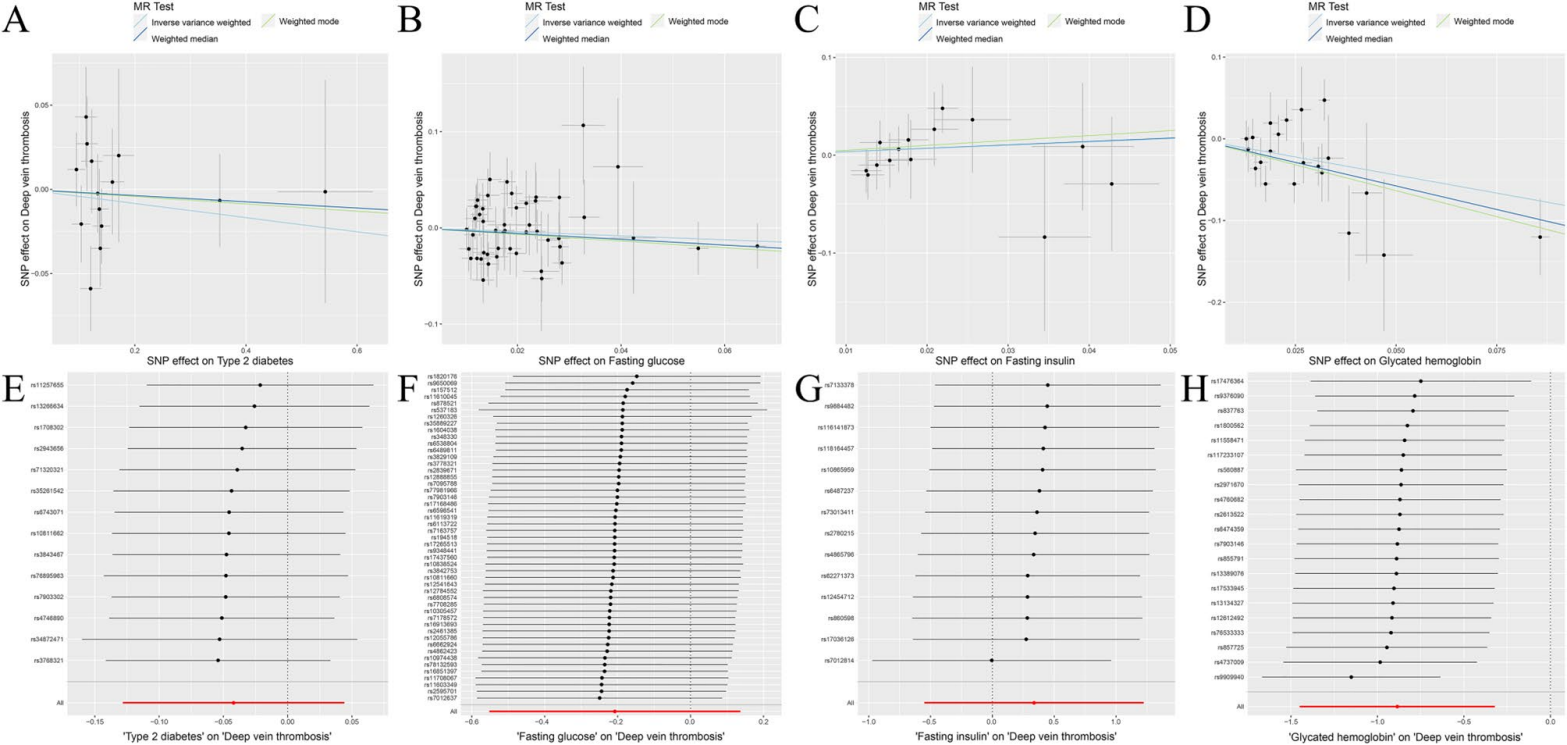
MR analysis results of the exposure (T2D, FG, FI, GH) and DVT. **A**: Scatter plot of T2D and DVT; **B**: Scatter plot of FG and DVT; **C**: Scatter plot of FI and DVT; **D**: Scatter plot of GH and DVT; **E**: Leave one out analysis of T2D and DVT; **F**: Leave one out analysis of FG and DVT; **G**: Leave one out analysis of FI and DVT; **H**: Leave one out analysis of GH and DVT

**Fig. 6:**
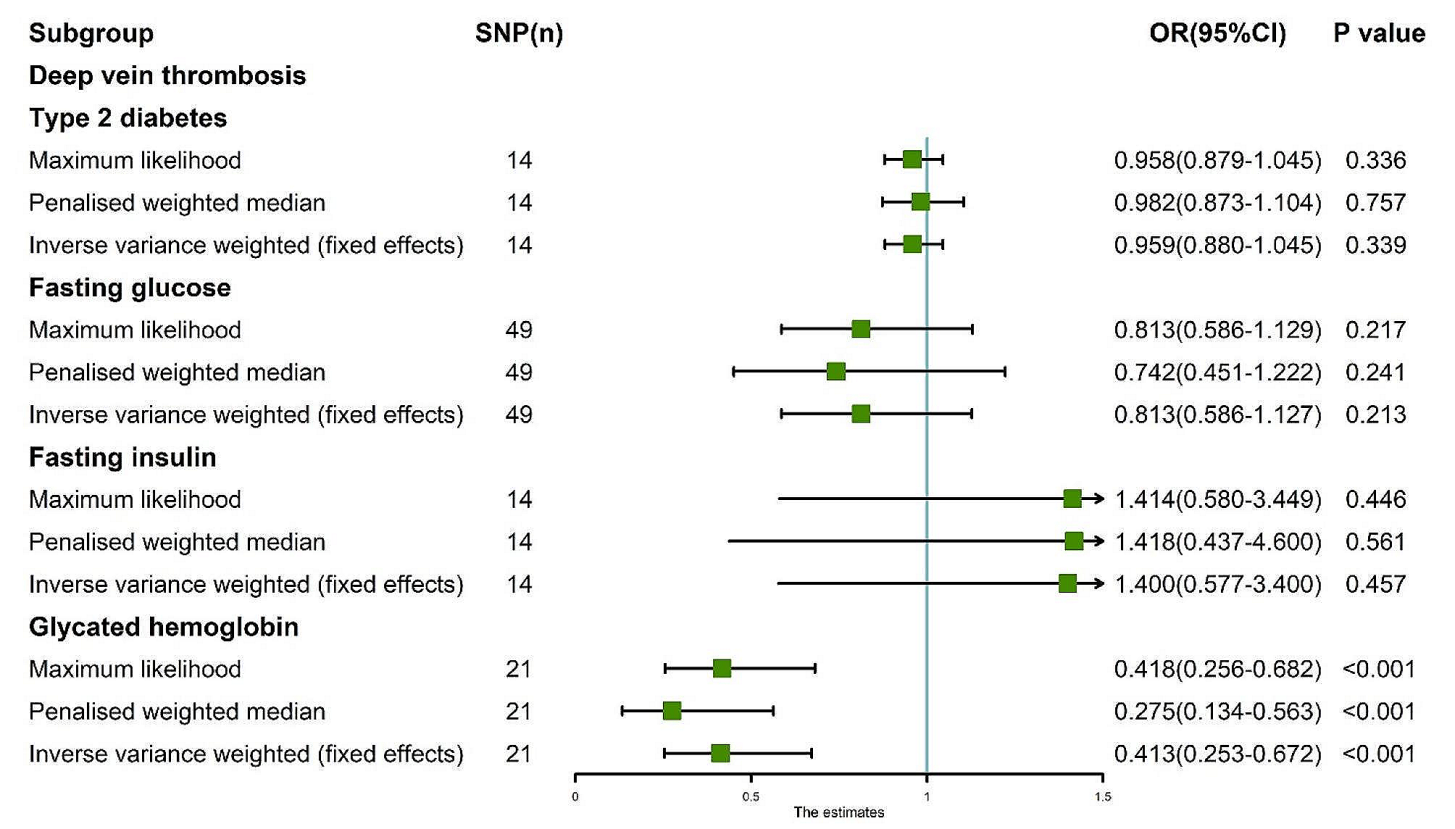
MR analysis results of the exposure (T2D, FG, FI, GH) and DVT. Three methods: maximum likelihood, penalised weighted median, and IVW (fixed effects)

The random-effects IVW analysis yielded findings indicating no genetic causal relationship between T2D and DVT (*P* = 0.339, OR 95% CI = 0.959 [0.880–1.045]). Results from Weighted Median and Weighted Mode analyses corroborated those obtained from the random-effects IVW model (Figs. 4 and 5A). Both Cochran’s Q statistic for MR-IVW and Rucker’s Q statistic for MR Egger demonstrated absence of heterogeneity (*P* > 0.05). Additionally, the intercept tests conducted in MR Egger and MR-PRESSO indicated no evidence of horizontal pleiotropy (*P* > 0.05). Notably, the MR-PRESSO analysis revealed no presence of outliers (Table 1). Furthermore, the “Leave One Out” analysis provided no indication that the MR analysis was influenced by any single SNP (Fig. 5E). Finally, results from Maximum Likelihood, Penalized Weighted Median, and IVW (fixed effects) analyses were consistent with those derived from the random-effects IVW model (*P* > 0.05) (Fig. 6).

The findings of the random-effects IVW analysis revealed no genetic causal association between FG and DVT (Supplementary Fig. 4A, 4C). Notably, both Cochran’s Q statistic of the MR-IVW and Rucker’s Q statistic of the MR Egger indicated heterogeneity within the MR analysis (*P* < 0.05). Furthermore, the intercept test of MR Egger indicated the absence of horizontal pleiotropy (*P* > 0.05), albeit the MR-PRESSO analysis suggested the presence of horizontal pleiotropy (*P* < 0.05), particularly identifying an outlier (rs10838693) and two potential outliers (rs1057394, rs2075423) (Supplementary Table 6). Subsequent “Leave one out” analysis confirmed that the MR analysis was not influenced by any single SNP (Supplementary Fig. 4C). Consequently, a secondary MR analysis was conducted post exclusion of rs10838693, wherein the random-effects IVW results still indicated an absence of genetic causal relationship between FG and DVT (Supplementary Fig. 4B, 4D), albeit with continued heterogeneity (*P* < 0.05). While the intercept test of MR Egger did not reveal horizontal pleiotropy (*P* > 0.05), the MR-PRESSO analysis reiterated the presence of horizontal pleiotropy (*P* < 0.05), identifying three potential outliers (rs189548, rs1057394, rs2075423) (Supplementary Table 6). Notably, the subsequent “Leave one out” analysis underscored the robustness of the MR analysis results against the influence of individual SNPs (Supplementary Fig. 4D). A third round of MR analysis ensued, following the elimination of the three potential outliers, which corroborated the earlier findings, revealing no significant genetic causal relationship between FG and DVT (*P* = 0.237, OR 95% CI = 0.813 [0.576–1.147]). Consistency was observed across the Weighted Median and Weighted Mode estimations with the random-effects IVW (Figs. 4 and 5B), along with the absence of heterogeneity, horizontal pleiotropy, or outliers in the MR analysis (Table 1). Furthermore, the “Leave one out” analysis further supported the robustness of the MR analysis results against the influence of individual SNPs (Fig. 5F). Additional analyses, including Maximum Likelihood, Penalised Weighted Median, and IVW with fixed effects, yielded consistent results with the random-effects IVW (*P* > 0.05) (Fig. 6).

The random-effects IVW analysis revealed a negative genetic causal relationship between FI and DVT (Supplementary Fig. 5A, 5B). Both Cochran’s Q statistic in MR-IVW and Rucker’s Q statistic in MR Egger indicated absence of heterogeneity (*P* > 0.05). Additionally, the intercept test of MR Egger and MR-PRESSO provided evidence against horizontal pleiotropy (*P* > 0.05). Furthermore, the MR-PRESSO analysis demonstrated no outliers (Supplementary Table 6). However, the “Leave one out” analysis revealed that the MR analysis was influenced by a single SNP (nine SNPs) (Supplementary Fig. 5B). Consequently, a secondary MR analysis was conducted after excluding these nine SNPs. Subsequent random-effects IVW analysis indicated no genetic causal relationship between FI and DVT (*P* = 0.457, OR 95% CI = 1.400 [0.577–3.401]). Consistency was observed in the Weighted Median and Weighted Mode analysis results compared to random-effects IVW (Figs. 4 and 5C). There was no observed heterogeneity, horizontal pleiotropy, or outliers (Table 1). Notably, the “Leave one out” analysis did not indicate any influence of a single SNP (Fig. 5G). Finally, results from Maximum Likelihood, Penalised Weighted Median, and IVW (fixed effects) analyses aligned with those from random-effects IVW (*P* > 0.05) (Fig. 6).

The random-effects IVW analysis revealed a negative genetic causal association between GH and DVT (*P* = 0.002, OR 95% CI = 0.413 [0.235–0.725]). Both Weighted Median and Weighted Mode analyses corroborated the findings of the random-effects IVW analysis (Figs. 4 and 5D). Examination of heterogeneity through Cochran’s Q statistic in MR-IVW and Rucker’s Q statistic in MR Egger indicated no significant heterogeneity (*P* > 0.05). Further assessment through the intercept test in MR Egger and MR-PRESSO revealed no evidence of horizontal pleiotropy (*P* > 0.05). Additionally, the MR-PRESSO analysis demonstrated the absence of outliers (Table 1). “Leave one out” analysis suggested that the MR analysis was not influenced by a single SNP (Fig. 5H). Finally, consistency in results was observed across Maximum Likelihood, Penalized Weighted Median, and IVW (fixed effects) analyses, all of which aligned with the findings of the random-effects IVW analysis (*P* < 0.05) (Fig. 6).

### MR analysis of exposure (T2D, FG, FI, GH) and PE

**Fig. 7:**
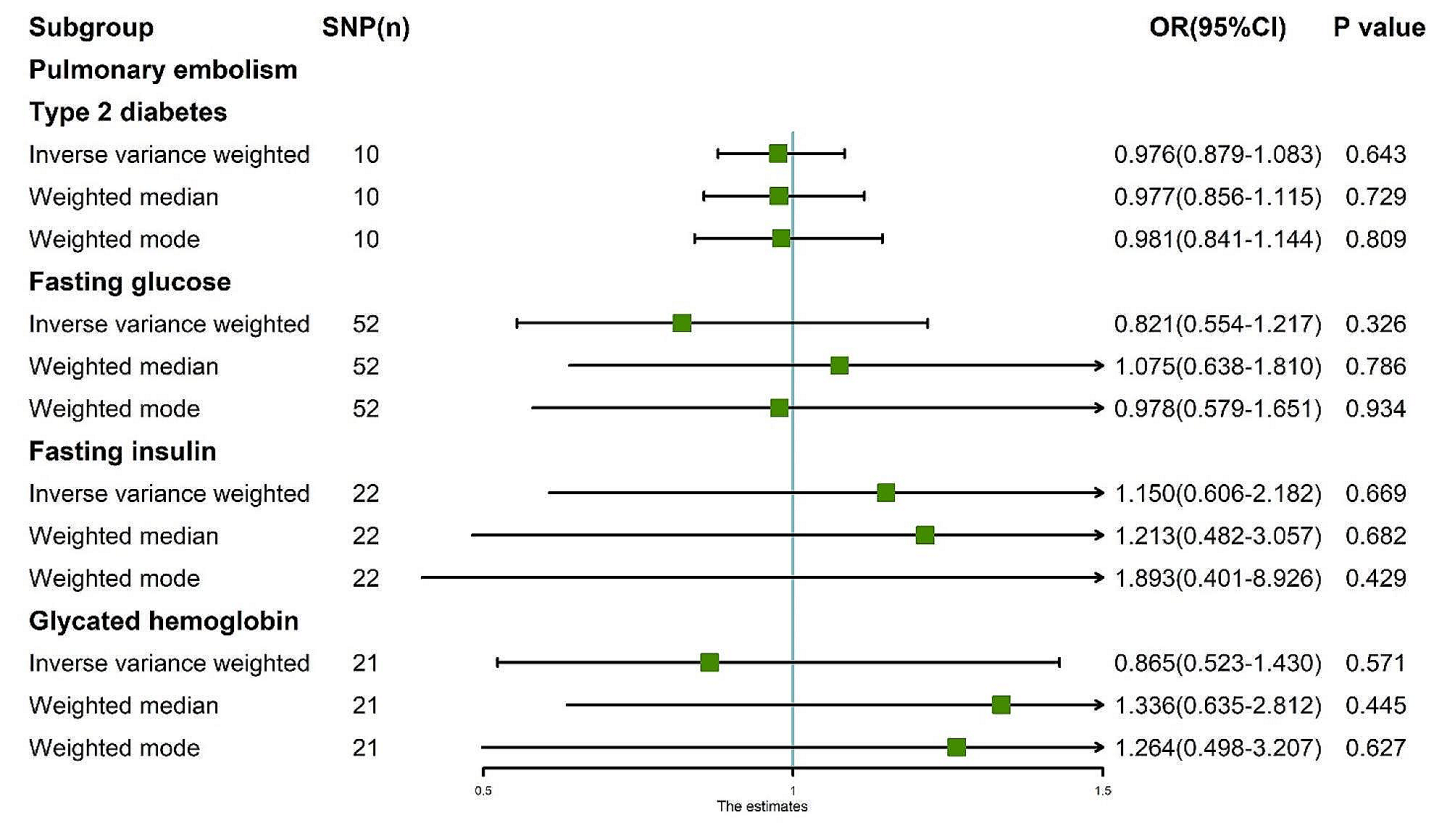
MR analysis results of the exposure (T2D, FG, FI, GH) and PE. Three methods: random-effects IVW, weighted median and weighted mode

**Fig. 8:**
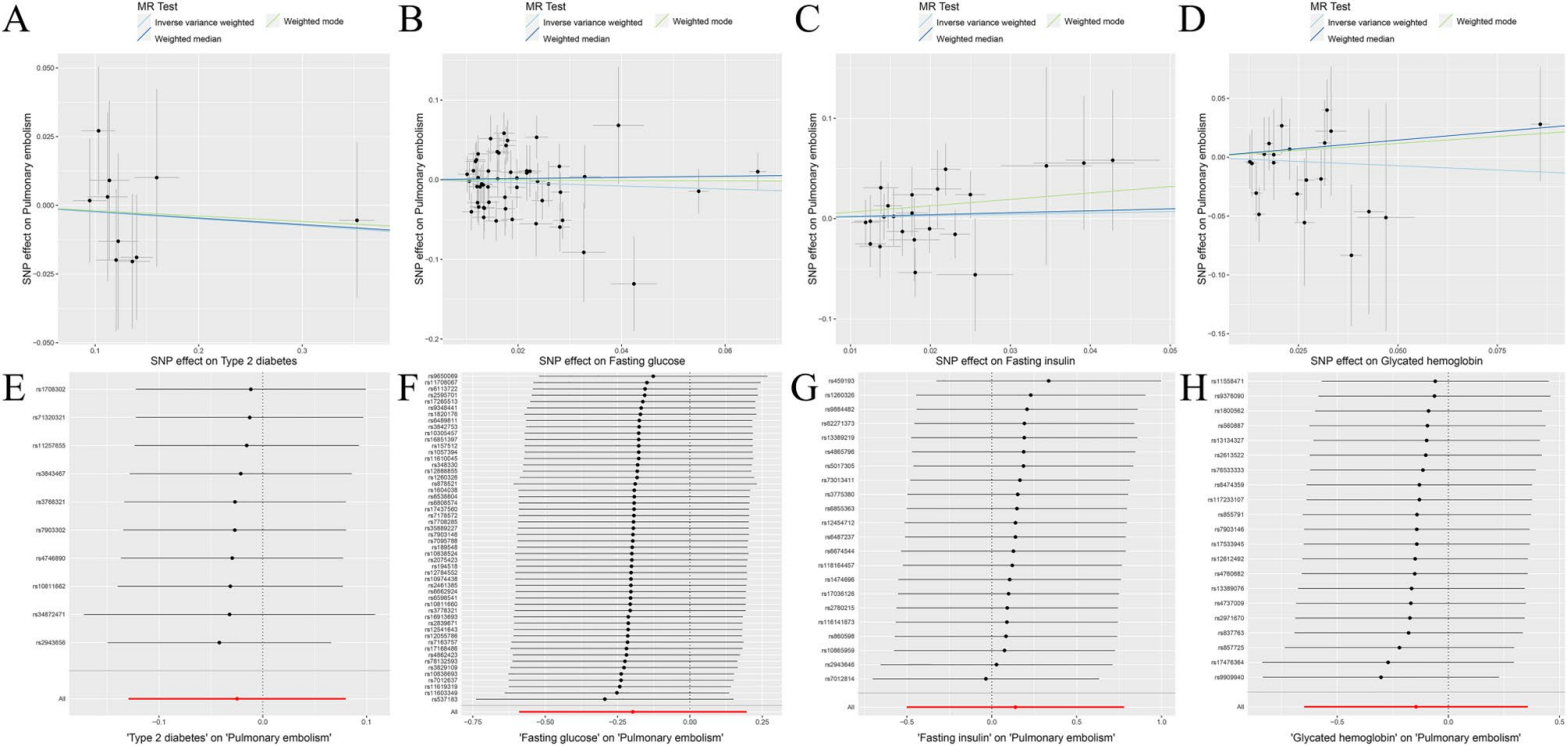
MR analysis results of the exposure (T2D, FG, FI, GH) and PE. **A**: Scatter plot of T2D and PE; **B**: Scatter plot of FG and PE; **C**: Scatter plot of FI and PE; **D**: Scatter plot of GH and PE; **E**: Leave one out analysis of T2D and PE; **F**: Leave one out analysis of FG and PE; **G**: Leave one out analysis of FI and PE; **H**: Leave one out analysis of GH and PE

**Fig. 9:**
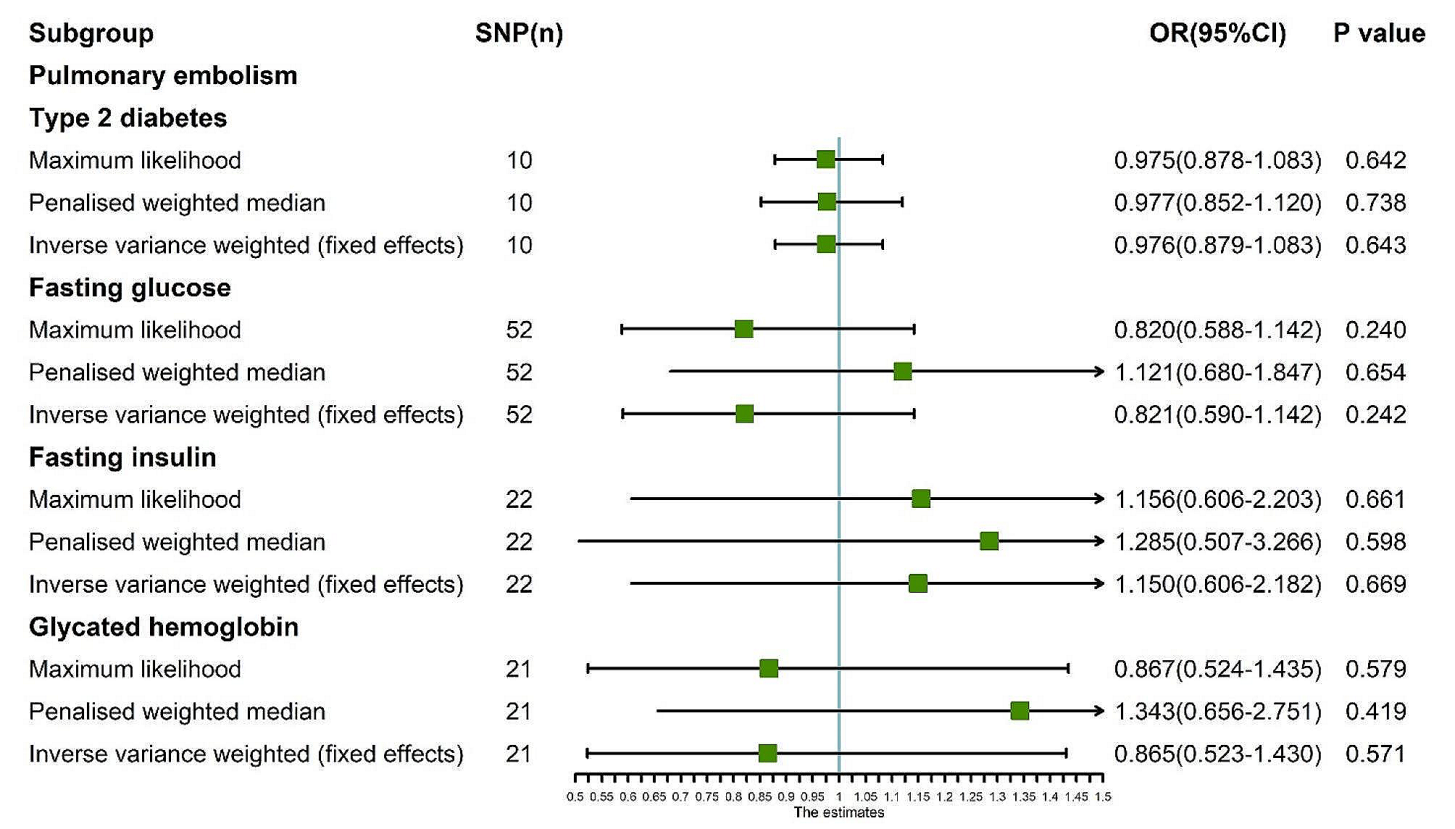
MR analysis results of the exposure (T2D, FG, FI, GH) and PE. Three methods: maximum likelihood, penalised weighted median, and IVW (fixed effects)

The random-effects IVW analysis revealed a negative genetic causal relationship between T2D and PE (Supplementary Fig. 6A, 6B). Both Cochran’s Q statistic of MR-IVW and Rucker’s Q statistic of MR Egger indicated absence of heterogeneity (*P* > 0.05). Moreover, the intercept test of MR Egger and MR-PRESSO affirmed the absence of horizontal pleiotropy (*P* > 0.05). MR-PRESSO analysis further corroborated these findings by revealing the absence of outliers (Supplementary Table 6). However, “Leave one out” analysis suggested that the MR analysis was influenced by a single SNP (four SNPs) (Supplementary Fig. 6B). Consequently, a secondary MR analysis was conducted after excluding the four aforementioned SNPs. The subsequent random-effects IVW analysis demonstrated that T2D did not exhibit a genetic causal relationship with PE (*P* = 0.643, OR 95% CI = 0.976 [0.879–1.083]). This observation was consistently supported by Weighted Median and Weighted Mode analyses (Figs. 7 and 8A). Furthermore, the MR analysis displayed no heterogeneity, horizontal pleiotropy, or outliers (Table 1), and “Leave one out” analysis indicated that the MR analysis was not influenced by a single SNP (Fig. 8E). Additionally, the results of Maximum Likelihood, Penalised Weighted Median, and IVW (Fixed Effects) analyses were in concordance with those of the random-effects IVW (*P* > 0.05) (Fig. 9).

The random-effects IVW analysis revealed no genetic causal association between FG and PE (Supplementary Fig. 7A, 7B). Both Cochran’s Q statistic for MR-IVW and Rucker’s Q statistic for MR Egger exhibited heterogeneity (*P* < 0.05). Moreover, the intercept test of MR Egger indicated an absence of horizontal pleiotropy (*P* > 0.05), yet the MR-PRESSO test detected evidence of horizontal pleiotropy (*P* < 0.05). Notably, the MR-PRESSO analysis identified a potential outlier, rs77981966, in the MR analysis (Supplementary Table 6). Subsequent “Leave one out” analysis suggested that the MR analysis was not solely influenced by a single SNP (Supplementary Fig. 7B). Consequently, a secondary round of MR analysis was conducted following the removal of rs77981966. The ensuing random-effects IVW analysis again failed to substantiate a genetic causal link between FG and PE (*P* = 0.326, OR 95% CI = 0.821 [0.554–1.217]). Consistency was observed across results obtained from Weighted Median and Weighted Mode analyses, aligning with the random-effects IVW (Figs. 7 and 8B). Furthermore, the secondary MR analysis exhibited indications of heterogeneity, and no horizontal pleiotropy, or outliers (Table 1), with “Leave one out” analysis affirming the robustness of the findings (Fig. 8F). Moreover, findings from Maximum Likelihood, Penalised Weighted Median, and Fixed Effects IVW analyses concurred with those of the random-effects IVW (*P* > 0.05) (Fig. 9).

The findings from the random-effects IVW analysis indicate no genetic causal association between FI and PE (Supplementary Fig. 8A, 8B). However, the examination of heterogeneity through Cochran’s Q statistic in MR-IVW and Rucker’s Q statistic in MR Egger suggests significant heterogeneity (*P* < 0.05). The intercept test within MR Egger, on the other hand, reveals no evidence of horizontal pleiotropy (*P* > 0.05); however, the MR-PRESSO analysis contradicts this, indicating the presence of horizontal pleiotropy (*P* < 0.05). Notably, the MR-PRESSO analysis identifies two outliers (rs75179845, rs7133378) and one potential outlier (rs459193) (Supplementary Table 6). Further analysis employing the “Leave One Out” method suggests that the MR results are not excessively influenced by any single SNPs (Supplementary Fig. 8B). Consequently, a secondary MR analysis was conducted following the exclusion of the identified two outliers. Subsequent random-effects IVW analysis still indicates no significant genetic causal link between FI and PE (*P* = 0.669, OR 95% CI = 1.150 [0.606–2.182]). Consistency in findings is observed through the Weighted Median and Weighted Mode methods (Figs. 7 and 8C). Moreover, this refined MR analysis demonstrates an absence of heterogeneity and horizontal pleiotropy (*P* > 0.05), as well as outlier effects (Table 1). Consistency is further corroborated by the “Leave One Out” analysis, which confirms the robustness of the MR results against single SNP influences (Fig. 8G). Additionally, findings from Maximum Likelihood, Penalized Weighted Median, and Fixed Effects IVW analyses align with those of the random-effects IVW analysis (*P* > 0.05) (Fig. 9).

The random-effects IVW analysis revealed no genetic causal association between GH and PE (*P* = 0.571, OR 95% CI = 0.865 [0.523–1.430]). Consistency in findings was observed across Weighted Median and Weighted Mode analyses (Figs. 7 and 8D). Further assessment using Cochran’s Q statistic for MR-IVW and Rucker’s Q statistic for MR Egger indicated absence of heterogeneity (*P* > 0.05). The application of intercept tests through MR Egger and MR-PRESSO demonstrated no evidence of horizontal pleiotropy (*P* > 0.05). Additionally, the MR-PRESSO analysis did not identify any outliers in the relationship between GH and PE (Table 1). The “Leave One Out” analysis provided assurance that the MR analysis was not unduly influenced by a single SNP (Fig. 8H). Finally, assessments conducted through Maximum Likelihood estimation, Penalised Weighted Median, and fixed-effects IVW analyses yielded consistent results with the random-effects IVW analysis (*P* > 0.05) (Fig. 9).

## Discussion

Our results showed that T2D, FG and GH had negative genetic causal relationship with VTE at the genetic level, while FI had no genetic causal relationship with VTE. GH had negative genetic causal relationship with DVT at the genetic level, while T2D, FG and FI had no genetic causal relationship with DVT. T2D, FG, FI and GH had no genetic causal relationship with PE. Our study provides new evidence on the pathogenesis, prevention and treatment of VTE, DVT and PE at the genetic level, and provides new ideas for research in this field.

One study found that T2D increased the risk of Khorana VTE in colorectal cancer patients [53]. In a study on inflammatory plasma markers and VTE risk assessment, T2D was found to be a risk factor for VTE [54]. The conclusion that T2D can increases the risk of VTE was also reached in another population-based cohort study [55]. FG is an important indicator of blood glucose levels and glucose tolerance in humans. Abnormal changes in FG are one of the most important manifestations of pre-diabetes and diabetes. Elevated blood glucose levels enhance blood clotting [56]. In addition, elevated FG can independently predict adult-specific VTE [57]. GH is an important indicator to assess the risk of diabetic complications. The study found that women with T2D with GH levels > 7% had a slightly higher risk of unprovoked VTE compared to women with GH levels > 6.5-7.0% [58]. These studies all found T2D, FG and GH to be risk factors for the development of VTE. Insulin resistance has been found to play an important role in the development of T2D [59–61]. And T2D, as well as high levels of FG and GH also increase the risk of the development of insulin resistance [62]. In addition, one study found that insulin resistance increases the risk of VTE [17]. We believe that it is because of T2D, as well as high levels of FG and GH, that insulin resistance occurs in the body, which in turn increases the risk of VTE. On the other hand, T2D and high levels of FG and GH can stimulate the release of insulin from pancreatic beta cells, and insulin can help improve vascular damage [63–65], therefore, the risk of thrombosis can be reduced. We are cautious to suggest that it is this pathway that allows a negative causal association between T2D, FG and GH and VTE at the genetic level. Hyperinsulinemia was found to inhibit fibrinolysis in a study by Michiel et al [56]. Also overweight patients with VTE have reduced insulin levels [66]. Increased risk of VTE in patients with insulin resistance [17]. Thomas et al. also found that insulin was associated with the development of VTE in pregnant women [67]. These studies found that insulin and VTE seem to maintain a strong association, but through our study, we found that there had no causal relationship between FI and VTE at the genetic level, which suggests that the pathway by which insulin affects VTE is not a genetic factor. And we consider that the reason why there had a relationship between the two in clinical observations may be due to the fact that insulin regulates blood glucose levels and affects thrombosis, which in turn leads to VTE.

In a study of preoperative DVT in total knee arthroplasty, patients with higher GH levels were found to be at increased risk of preoperative DVT [23]. Another study found that the incidence of DVT was significantly higher in diabetic patients than in non-diabetic patients within 3d after unicompartmental knee arthroplasty (UKA), and was proportional to GH concentration [68]. GH assesses serum glucose levels, and hyperglycemia induces insulin release, which ameliorates vascular endothelial cell injury [63, 64]. And vascular endothelial injury is an important part of the development of DVT [69, 70]. We consider that it may be the presence of this pathway that causes a negative causal association between GH and DVT at the genetic level. In a population-based cohort study, the risk of DVT was found to be higher in the group of patients with T2D than in the control group [55]. Another study found that T2D can increases risk of DVT in pulmonary embolism and chronic obstructive pulmonary disease (PE-COPD) [71]. Postoperative FG as a risk factor for DVT in a study of risk factors for lower extremity DVT in elderly knee replacements [72]. Elevated plasma insulin levels and severe traumatic brain injury (TBI) can increase the risk of DVT [56]. There was an association between T2D, FG and FI and DVT in these studies. T2D, FG and FI were risk factors for DVT, but no significant genetic-level causal relationship was found between them in our study. T2D and abnormal FG and FI may be involved in the development of DVT by damaging endothelium and altering blood status [73]. This may be the reason why T2D, FG and FI are risk factors for DVT, but there is no causal relationship between them and DVT at the genetic level.

In a population-based cohort study, the risk of PE was found to be higher in the T2D patient group than in the control group [55]. A study conducted in Spain found a higher incidence of PE in patients with T2D than in the general population [74]. Another study found that patients with a history of T2D was associated with the risk of developing PE [75]. In one study, patients with higher FG, FI, had a higher risk of PE [76]. However, no association was found between GH ≥ 7% and the risk of DVT or PE [77]. Our study results showed that neither T2D nor glycemic traits are causally associated with PE at the genetic level. It has been shown that T2D and glycemic traits can increase the risk of VTE [78, 79]. An important factor in the occurrence of PE is the formation of PE after the venous thrombus is dislodged and enters the pulmonary artery through the circulation [80]. We cautiously consider that it is may because T2D and glycemic traits increase the risk of VTE and embolism shedding to form PE that an association between both T2D and glycemic traits and PE has been observed in a large number of studies. Through our study, new evidence is provided at the genetic level, confirming that they are not genetically causally related, which can provide new ideas for research in this field.

This study is the first to analyze the genetic causal relationship between T2D, FG, FI, GH and VTE, DVT, PE using the MR analysis based on the large-scale GWAS summary data. However, some limitations are inevitable. First, the population of our study is European population, and the applicability of our findings when extended to other populations requires further validation. Second, our study did not examine men and women separately, and our findings may differ somewhat when applied to a single-sex analysis. Finally, our study only explored causality at the genetic level, and the complexity of the disease should be fully considered when our conclusions are applied to the predictive assessment of diseases such as VTE, DVT, and PE.

## Conclusion

In conclusion, our study found that T2D, FG and GH had negative genetic causal relationship with VTE at the genetic level, while FI had no genetic causal relationship with VTE. GH had negative genetic causal relationship with DVT at the genetic level, while T2D, FG and FI had no genetic causal relationship with DVT. T2D, FG, FI and GH had no genetic causal relationship with PE. Our results can provide new ideas for the prevention and treatment of VTE, DVT and PE, and facilitate the study of the diseases. However, considering the diversity of disease etiology, we need to further study the relationship between T2D, blood glucose characteristics and VTE, DVT and PE, so that we can understand the relationship between them more clearly and accurately.

### Electronic supplementary material

Below is the link to the electronic supplementary material.

Supplementary Material 1

## Data Availability

.Publicly available datasets were analyzed in this study. This data can be found here: IEU Open GWAS database (https://gwas.mrcieu.ac.uk/).
