# Supplementary material for: The genetic causal relationship between type 2 diabetes, glycemic traits and venous thromboembolism, deep vein thrombosis, pulmonary embolism: a two-sample Mendelian randomization study
Source: Thromb J. 2024 Mar 29;22:33. doi: 10.1186/s12959-024-00600-z (PMC10979561; doi:10.1186/s12959-024-00600-z)
Supplement: Supplementary file 1 — Supplementary Material 1 [file 12959_2024_600_MOESM1_ESM.docx]

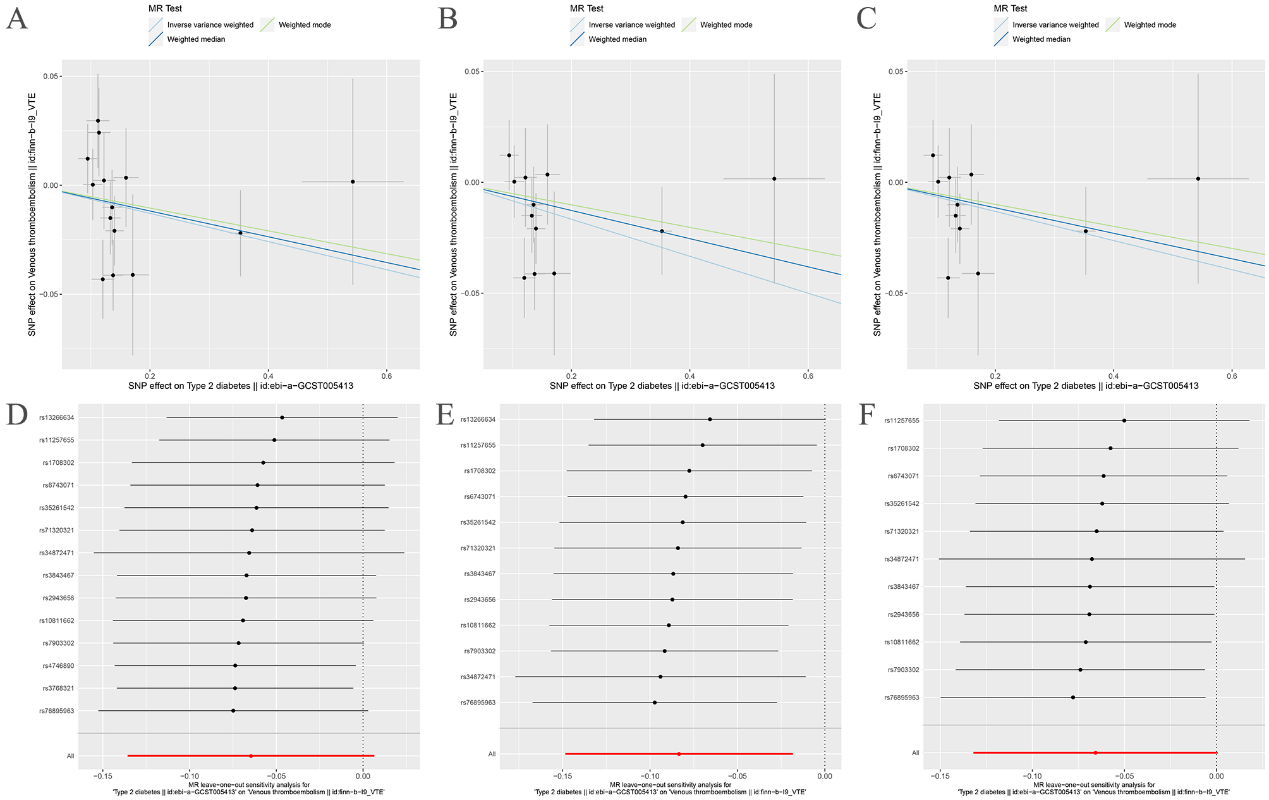


**Supplementary Figure 1:** MR analysis of the first three rounds of type 2 diabetes and venous thromboembolism.


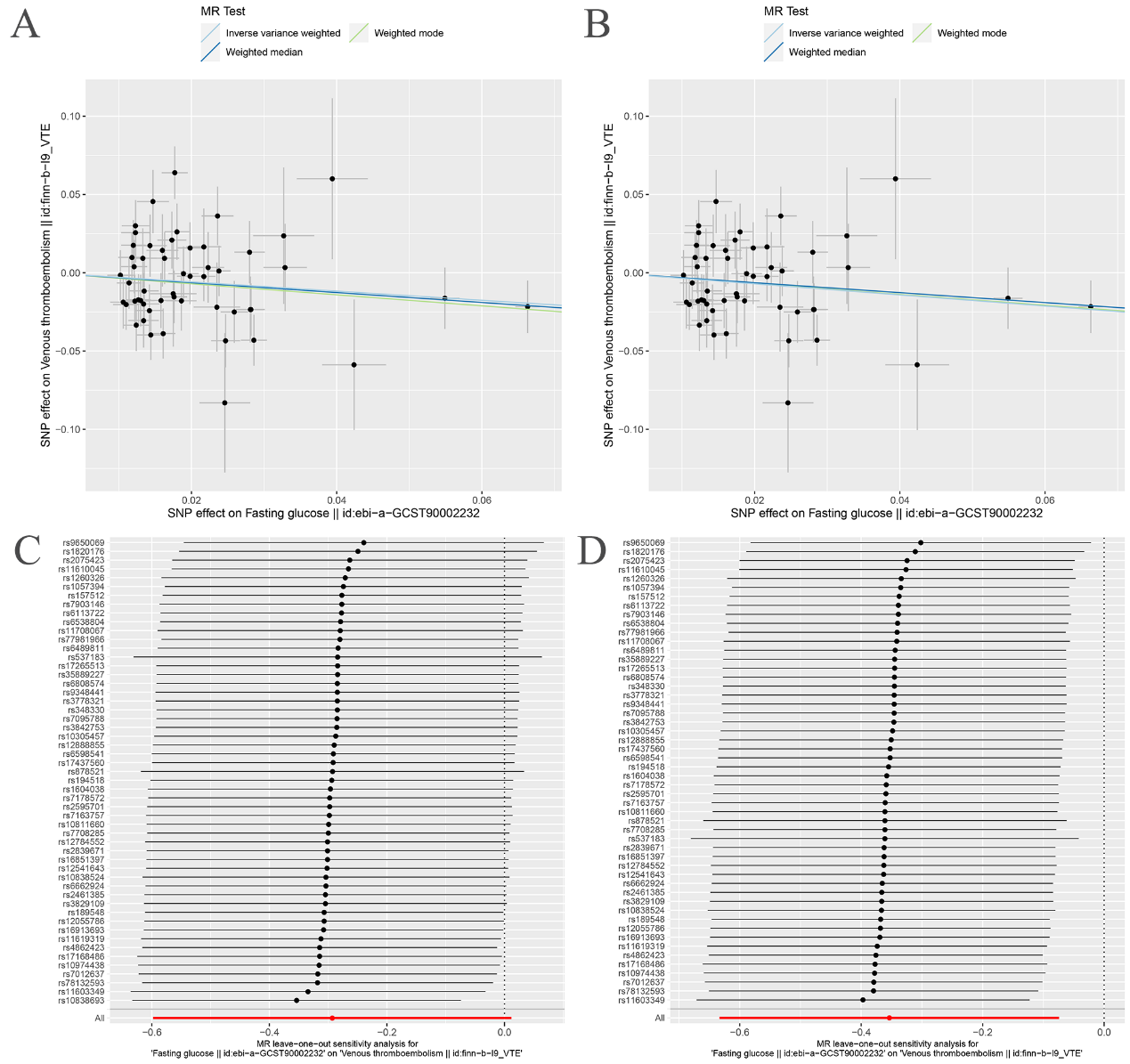


**Supplementary Figure 2:** MR analysis of the first two rounds of fasting glucose and venous thromboembolism.


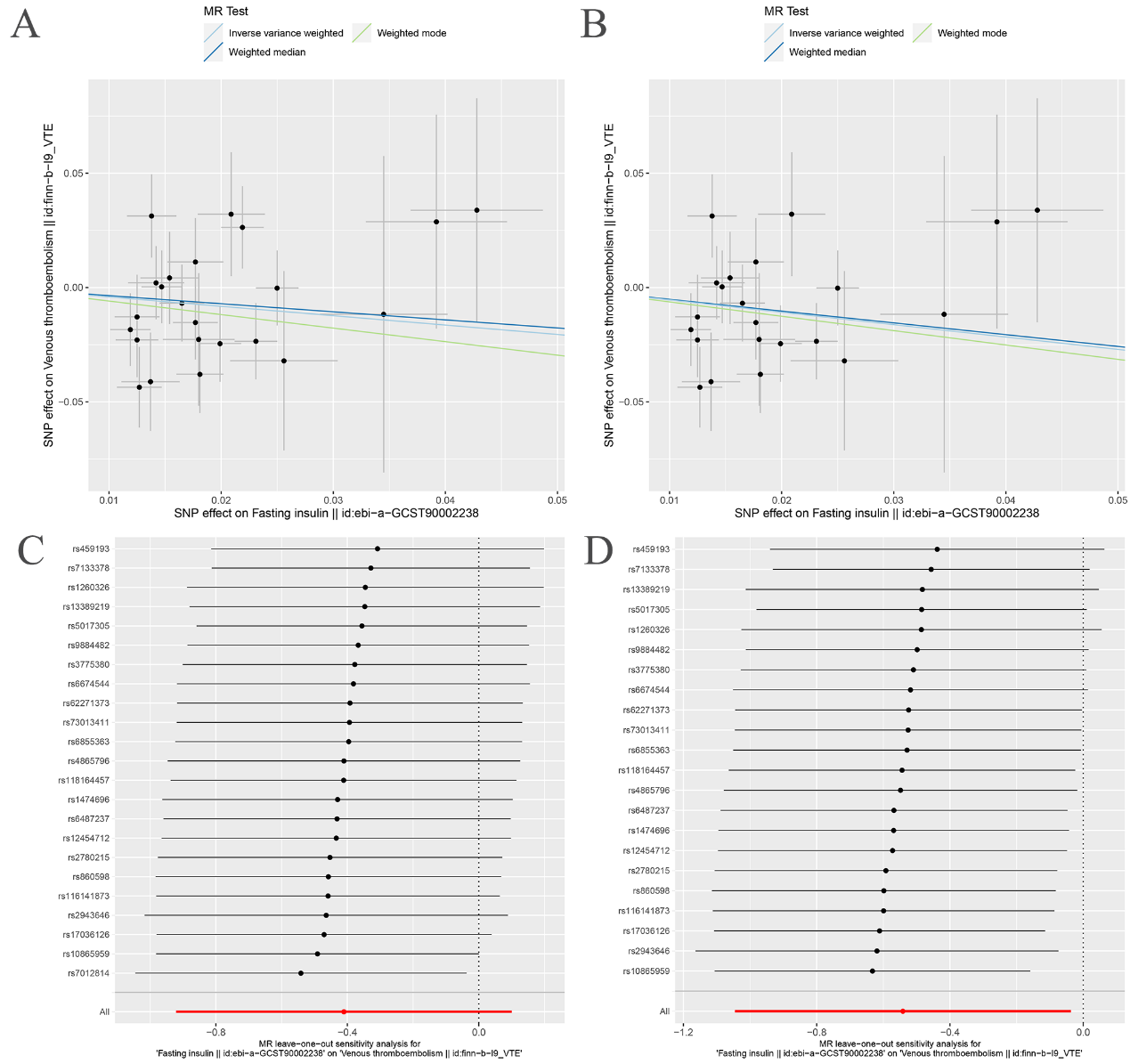


**Supplementary Figure 3:** MR analysis of the first two rounds of fasting insulin and venous thromboembolism.


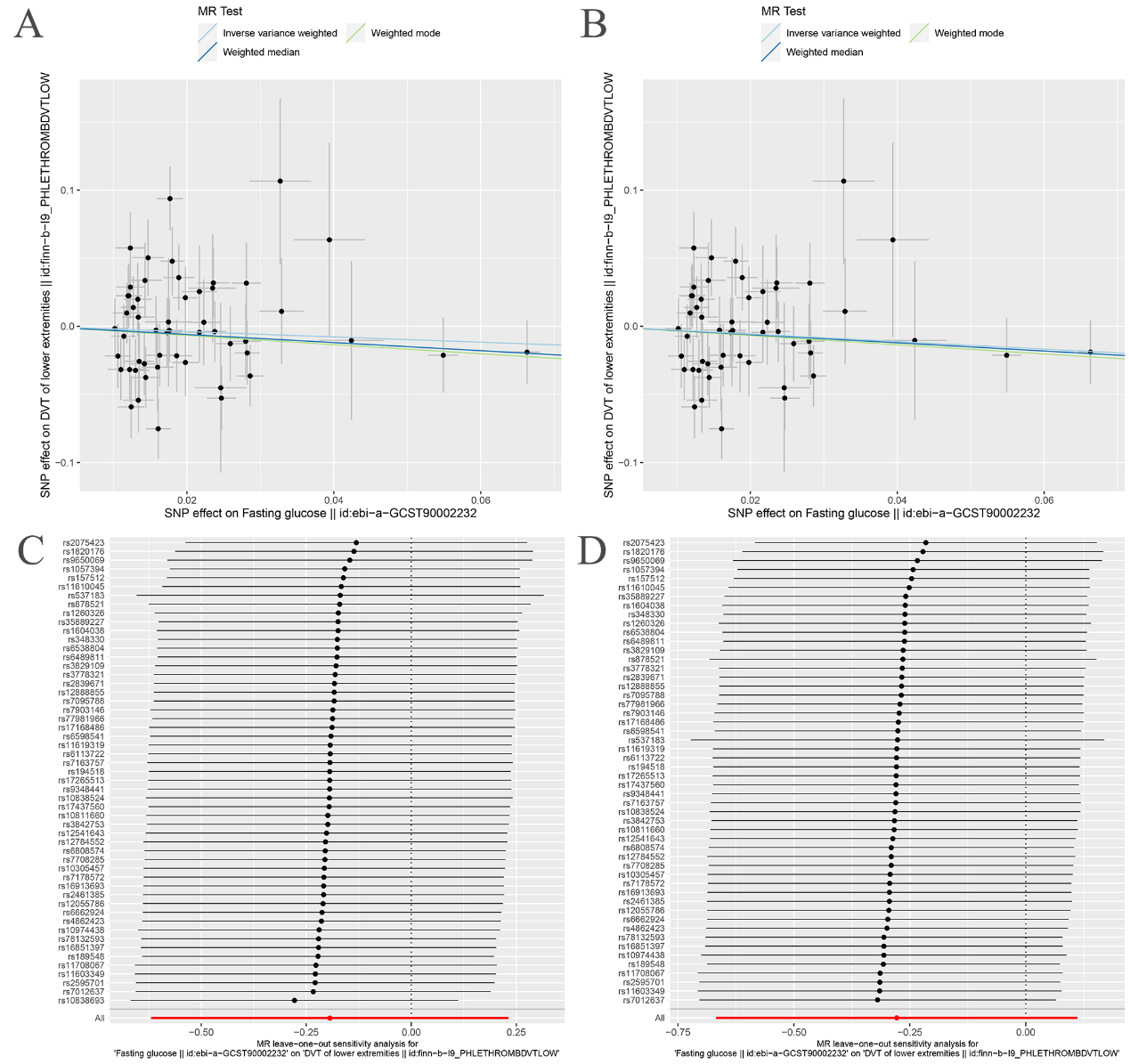


**Supplementary Figure 4:** MR analysis of the first two rounds of fasting glucose and deep vein thrombosis.


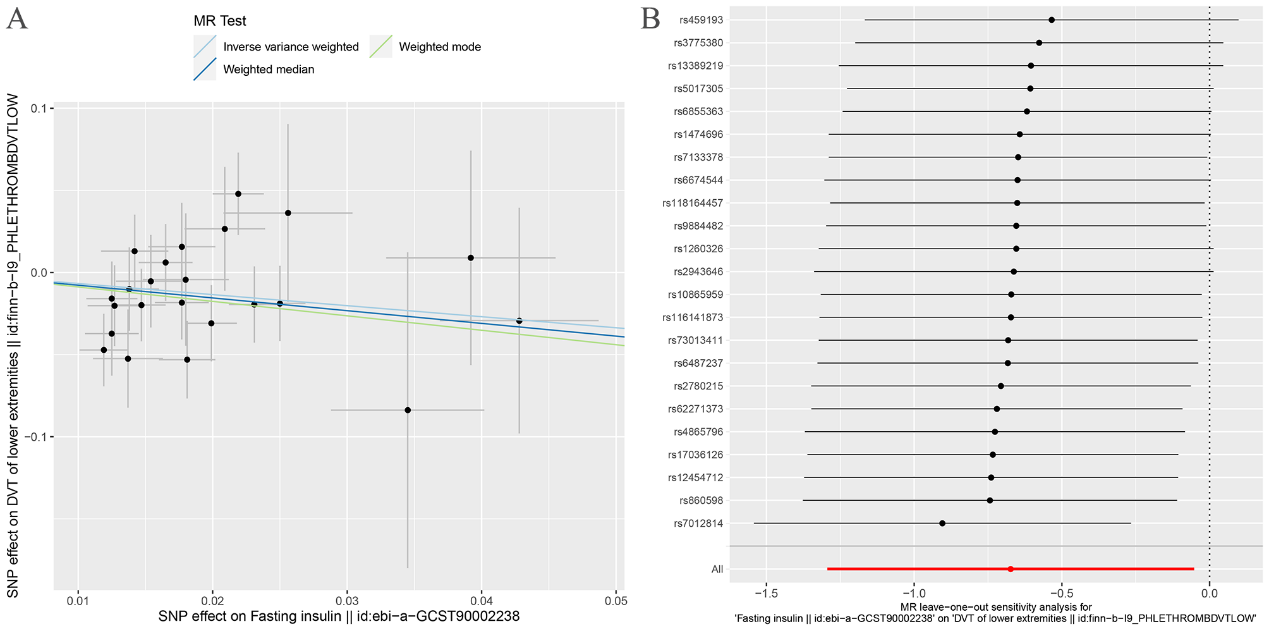


**Supplementary Figure 5:** First-round MR analysis of fasting insulin and deep vein thrombosis.


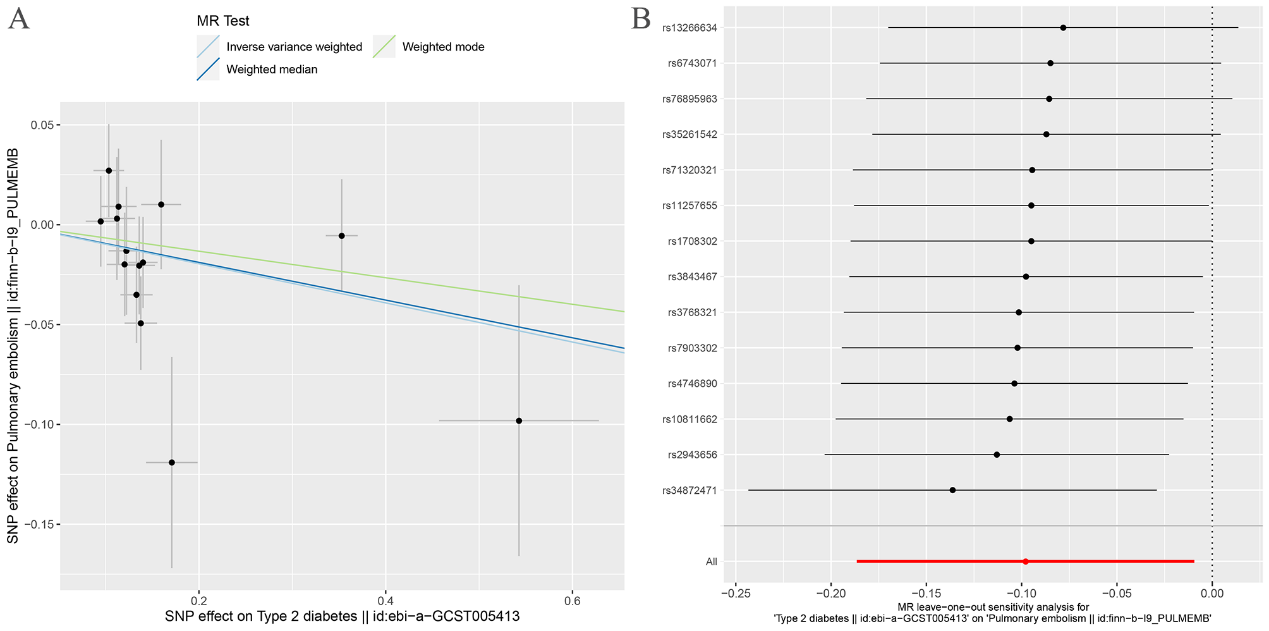


**Supplementary Figure 6:** First-round MR analysis of type 2 diabetes and pulmonary embolism.


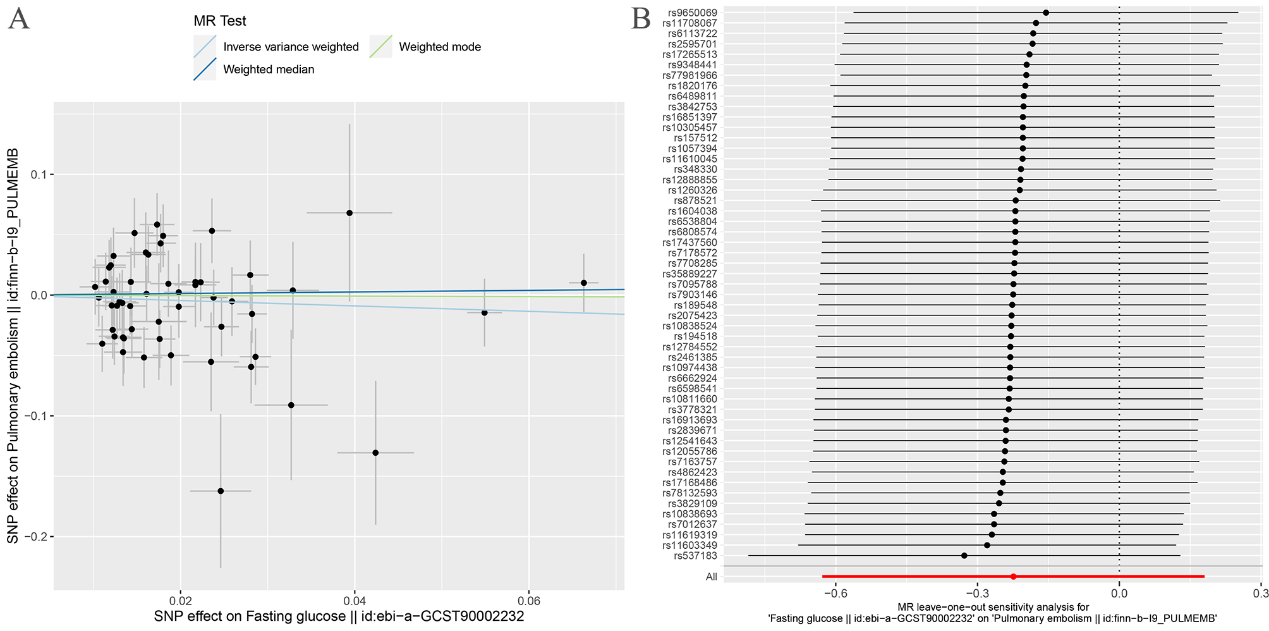


**Supplementary Figure 7:** First-round MR analysis of fasting glucose and pulmonary embolism.


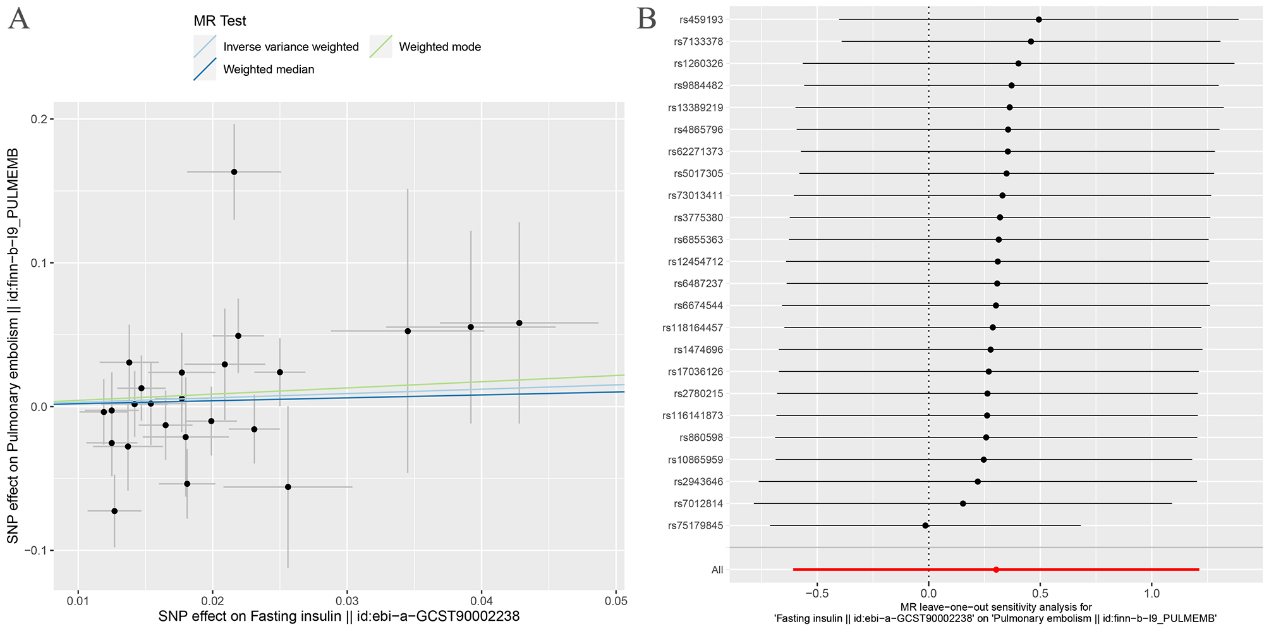


**Supplementary Figure 8:** First-round MR analysis of fasting insulin and pulmonary embolism.

**Supplementary Table 1:** The GWAS summary data used in this study.

|  | **GWAS ID** | **Year** | **Trait** | **Sample size** | **Number of SNPs** | **Population** | **PMID** |
| --- | --- | --- | --- | --- | --- | --- | --- |
| exposure | ebi-a-GCST005413 | 2018 | Type 2 diabetes | 70,127 | 14,277,791 | European | 29358691 |
| exposure | ebi-a-GCST90002232 | 2021 | Fasting glucose | 200,622 | 31,008,728 | European | 34059833 |
| exposure | ebi-a-GCST90002238 | 2021 | Fasting insulin | 151,013 | 29,664,438 | European | 34059833 |
| exposure | ebi-a-GCST90002244 | 2021 | Glycated hemoglobin levels | 146,806 | 30,649,064 | European | 34059833 |
| outcome | finn-b-I9_PHLETHROMBDVTLOW | 2021 | DVT of lower extremities | 218,792 | 16,380,409 | European | NA |
| outcome | finn-b-I9_VTE | 2021 | Venous thromboembolism | 194,604 | 16,380,466 | European | NA |
| outcome | finn-b-I9_PULMEMB | 2021 | Pulmonary embolism | 218,413 | 16,380,466 | European | NA |

**Supplementary Table 2:** The instrumental variables of MR analysis between type 2 diabetes and three outcomes (venous thromboembolism, deep vein thrombosis and pulmonary embolism).

|  | **SNP** | **beta.T2D** | **eaf.T2D** | **pval.T2D** | **pval.VTE** | **pval.DVT** | **pval.PE** | **R2** | **F** |
| --- | --- | --- | --- | --- | --- | --- | --- | --- | --- |
| 1 | rs10811662 | -0.1595 | 0.1706 | 1.00E-13 | 0.878 | 0.8886 | 0.7545 | 0.007199 | 508.51674 |
| 2 | rs11257655 | 0.1204 | 0.2097 | 3.74E-10 | 0.01732 | 0.01903 | 0.4437 | 0.004805 | 338.56181 |
| 3 | rs13266634 | -0.1377 | 0.3047 | 3.01E-15 | 0.01128 | 0.1224 | 0.03517 | 0.008034 | 567.96129 |
| 4 | rs1708302 | -0.1401 | 0.4916 | 2.93E-19 | 0.1891 | 0.3261 | 0.4082 | 0.009811 | 694.83 |
| 5 | rs2943656 | 0.1034 | 0.6344 | 2.74E-10 | 0.987 | 0.3629 | 0.2442 | 0.00496 | 349.52043 |
| 6 | rs34872471 | 0.3529 | 0.701 | 1.15E-94 | 0.2654 | 0.8107 | 0.847 | 0.052206 | 3862.6162 |
| 7 | rs35261542 | 0.1331 | 0.2765 | 1.66E-14 | 0.3741 | 0.9191 | 0.1461 | 0.007088 | 500.58965 |
| 8 | rs3768321 | 0.1121 | 0.1965 | 9.02E-09 | 0.1682 | 0.1489 | 0.9187 | 0.003968 | 279.37614 |
| 9 | rs3843467 | 0.1223 | 0.2032 | 1.90E-10 | 0.922 | 0.5904 | 0.6822 | 0.004843 | 341.30088 |
| 10 | rs4746890 | -0.1138 | 0.7833 | 4.41E-09 | 0.2313 | 0.3354 | 0.7524 | 0.004396 | 309.66206 |
| 11 | rs6743071 | -0.1709 | 0.904 | 5.77E-10 | 0.266 | 0.6967 | 0.02396 | 0.005069 | 357.30069 |
| 12 | rs71304101 | -0.2013 | 0.1222 | 4.39E-16 | 0.537499 | 0.3225 | 0.7603 | 0.008693 | 614.96324 |
| 13 | rs71320321 | 0.136 | 0.3184 | 5.05E-16 | 0.555801 | 0.6229 | 0.4065 | 0.008028 | 567.52362 |
| 14 | rs76895963 | -0.5427 | 0.9794 | 2.27E-10 | 0.973 | 0.9835 | 0.1478 | 0.011884 | 843.41646 |
| 15 | rs7903302 | -0.0948 | 0.5756 | 3.52E-09 | 0.4414 | 0.5939 | 0.9396 | 0.004391 | 309.26217 |
| 16 | rs9268835 | 0.1276 | 0.2914 | 1.37E-11 | 0.006361 | 0.04481 | 0.003267 | 0.006724 | 474.7062 |
| Confounding | |  |  |  |  |  |  |  |  |
| 1 | rs9268835 | Malignant neoplasm of anus and anal canal | | | | | | | |
| 2 | rs71304101 | Malignant neoplasm of other and unspecified parts of tongue | | | | | | | |

**Supplementary Table 3:** The instrumental variables of MR analysis between fasting glucose and three outcomes (venous thromboembolism, deep vein thrombosis and pulmonary embolism).

|  | **SNP** | **beta.FG** | **eaf.FG** | **pval.FG** | **pval.VTE** | **pval.DVT** | **pval.PE** | **R2** | **F** |
| --- | --- | --- | --- | --- | --- | --- | --- | --- | --- |
| 1 | rs39713 | -0.0169 | 0.062 | 1.77E-08 | 0.3864 | 0.760801 | 0.274 | 3.32E-05 | 6.664792 |
| 2 | rs4760278 | -0.011 | 0.181 | 3.33E-08 | 0.3871 | 0.4195 | 0.549301 | 3.59E-05 | 7.197268 |
| 3 | rs17270243 | 0.0104 | 0.24 | 3.62E-08 | 0.2596 | 0.8819 | 0.05129 | 3.95E-05 | 7.916129 |
| 4 | rs2657879 | 0.0119 | 0.202 | 7.33E-09 | 0.066181 | 0.2346 | 0.1673 | 4.57E-05 | 9.15951 |
| 5 | rs12898997 | -0.0098 | 0.598 | 4.64E-09 | 0.3476 | 0.4912 | 0.762 | 4.62E-05 | 9.264109 |
| 6 | rs896854 | -0.0099 | 0.542 | 5.61E-09 | 0.2405 | 0.3034 | 0.2892 | 4.87E-05 | 9.762488 |
| 7 | rs7095788 | -0.0106 | 0.358 | 1.98E-09 | 0.2649 | 0.3558 | 0.9366 | 5.16E-05 | 10.36231 |
| 8 | rs194518 | 0.0102 | 0.52 | 8.76E-09 | 0.921 | 0.9439 | 0.7674 | 5.19E-05 | 10.4201 |
| 9 | rs12888855 | -0.0135 | 0.189 | 6.02E-12 | 0.5684 | 0.3651 | 0.2205 | 5.59E-05 | 11.2093 |
| 10 | rs2302593 | -0.0106 | 0.495 | 5.67E-10 | 0.03798 | 0.05935 | 0.05093 | 5.62E-05 | 11.27034 |
| 11 | rs2238435 | -0.0112 | 0.619 | 3.82E-09 | 0.05804 | 0.4972 | 0.5561 | 5.92E-05 | 11.87084 |
| 12 | rs6598541 | -0.0114 | 0.648 | 4.12E-12 | 0.6944 | 0.7536 | 0.635199 | 5.93E-05 | 11.89481 |
| 13 | rs17437560 | -0.0175 | 0.11 | 3.33E-08 | 0.692901 | 0.9453 | 0.6511 | 6.00E-05 | 12.03065 |
| 14 | rs6489811 | 0.011 | 0.512 | 3.27E-09 | 0.2102 | 0.1619 | 0.083171 | 6.05E-05 | 12.13125 |
| 15 | rs189548 | -0.0123 | 0.723 | 2.81E-09 | 0.1806 | 0.03029 | 0.9245 | 6.06E-05 | 12.15791 |
| 16 | rs77981966 | -0.0246 | 0.055 | 1.58E-14 | 0.06195 | 0.4689 | 0.01077 | 6.29E-05 | 12.62107 |
| 17 | rs7178572 | 0.0121 | 0.679 | 7.09E-10 | 0.8175 | 0.3413 | 0.723 | 6.38E-05 | 12.80494 |
| 18 | rs6662924 | 0.0143 | 0.198 | 3.34E-10 | 0.3842 | 0.2216 | 0.7009 | 6.49E-05 | 13.02999 |
| 19 | rs10305457 | 0.0235 | 0.065 | 1.21E-14 | 0.4421 | 0.4802 | 0.1755 | 6.71E-05 | 13.46772 |
| 20 | rs12055786 | 0.012 | 0.384 | 1.17E-11 | 0.2716 | 0.3142 | 0.2778 | 6.81E-05 | 13.6681 |
| 21 | rs348330 | -0.0122 | 0.631 | 3.04E-10 | 0.2803 | 0.173 | 0.2273 | 6.93E-05 | 13.90624 |
| 22 | rs12541643 | 0.0118 | 0.479 | 4.51E-09 | 0.535499 | 0.6574 | 0.3124 | 6.95E-05 | 13.9435 |
| 23 | rs78132593 | -0.0147 | 0.203 | 2.60E-10 | 0.02444 | 0.07307 | 0.07464 | 6.99E-05 | 14.0289 |
| 24 | rs2839671 | -0.016 | 0.164 | 8.38E-14 | 0.5358 | 0.3513 | 0.2869 | 7.02E-05 | 14.08396 |
| 25 | rs157512 | -0.0134 | 0.27 | 5.43E-10 | 0.077469 | 0.024 | 0.1574 | 7.08E-05 | 14.2014 |
| 26 | rs1057394 | -0.0124 | 0.625 | 1.91E-12 | 0.04198 | 0.0101 | 0.1482 | 7.21E-05 | 14.46073 |
| 27 | rs3842753 | -0.0134 | 0.72 | 2.84E-09 | 0.3091 | 0.802 | 0.09264 | 7.24E-05 | 14.52566 |
| 28 | rs4862423 | 0.0123 | 0.401 | 4.45E-10 | 0.06654 | 0.2042 | 0.1671 | 7.27E-05 | 14.582 |
| 29 | rs7708285 | -0.0133 | 0.686 | 1.25E-09 | 0.6265 | 0.4526 | 0.8148 | 7.62E-05 | 15.28954 |
| 30 | rs6808574 | 0.0127 | 0.606 | 7.21E-14 | 0.2918 | 0.5385 | 0.7059 | 7.70E-05 | 15.45304 |
| 31 | rs35889227 | -0.013 | 0.617 | 3.37E-10 | 0.2794 | 0.1592 | 0.8181 | 7.99E-05 | 16.02542 |
| 32 | rs17265513 | 0.0158 | 0.204 | 5.10E-14 | 0.3164 | 0.9116 | 0.04096 | 8.11E-05 | 16.2666 |
| 33 | rs507666 | 0.0164 | 0.189 | 6.99E-17 | 8.84E-53 | 4.90E-38 | 1.13E-17 | 8.25E-05 | 16.54285 |
| 34 | rs16913693 | -0.0394 | 0.029 | 2.82E-16 | 0.2422 | 0.3728 | 0.3533 | 8.74E-05 | 17.5409 |
| 35 | rs16851397 | -0.0327 | 0.045 | 1.26E-12 | 0.587 | 0.080231 | 0.1432 | 9.19E-05 | 18.43977 |
| 36 | rs6538804 | -0.0142 | 0.377 | 9.41E-14 | 0.1456 | 0.2326 | 0.704001 | 9.47E-05 | 19.00428 |
| 37 | rs3778321 | -0.0186 | 0.176 | 3.16E-17 | 0.3502 | 0.416 | 0.727601 | 0.0001 | 20.13324 |
| 38 | rs11610045 | 0.0144 | 0.454 | 3.26E-13 | 0.01292 | 0.091821 | 0.2178 | 0.000103 | 20.62635 |
| 39 | rs3829109 | -0.0163 | 0.276 | 1.09E-15 | 0.5756 | 0.3599 | 0.1582 | 0.000106 | 21.30459 |
| 40 | rs11619319 | 0.0173 | 0.234 | 3.41E-20 | 0.2509 | 0.8576 | 0.02482 | 0.000107 | 21.52721 |
| 41 | rs2461385 | -0.0217 | 0.852 | 2.73E-19 | 0.499001 | 0.4548 | 0.8076 | 0.000119 | 23.8274 |
| 42 | rs2075423 | -0.0161 | 0.376 | 3.18E-21 | 0.01543 | 0.000746 | 0.9616 | 0.000122 | 24.40514 |
| 43 | rs9348441 | 0.0176 | 0.272 | 4.40E-20 | 0.3601 | 0.9027 | 0.1318 | 0.000123 | 24.61405 |
| 44 | rs174583 | -0.0168 | 0.375 | 3.37E-22 | 0.000851 | 0.001441 | 0.01453 | 0.000132 | 26.54554 |
| 45 | rs10838693 | 0.0177 | 0.314 | 3.44E-23 | 0.000149 | 6.32E-05 | 0.07485 | 0.000135 | 27.0809 |
| 46 | rs10811660 | -0.0223 | 0.165 | 7.94E-25 | 0.8788 | 0.924 | 0.7399 | 0.000137 | 27.49438 |
| 47 | rs12784552 | -0.0329 | 0.076 | 2.86E-31 | 0.9029 | 0.7755 | 0.9224 | 0.000152 | 30.50335 |
| 48 | rs2595701 | -0.0189 | 0.683 | 4.48E-19 | 0.9774 | 0.1369 | 0.04418 | 0.000155 | 31.03666 |
| 49 | rs11603349 | -0.0236 | 0.168 | 3.12E-25 | 0.05214 | 0.2191 | 0.04701 | 0.000156 | 31.24125 |
| 50 | rs1604038 | -0.0198 | 0.288 | 4.47E-28 | 0.9006 | 0.2884 | 0.7103 | 0.000161 | 32.26093 |
| 51 | rs7012637 | -0.018 | 0.47 | 9.75E-25 | 0.1456 | 0.05645 | 0.05695 | 0.000161 | 32.38867 |
| 52 | rs10974438 | 0.0198 | 0.38 | 9.85E-31 | 0.3321 | 0.3558 | 0.921 | 0.000185 | 37.06723 |
| 53 | rs6113722 | -0.0424 | 0.066 | 7.66E-25 | 0.1582 | 0.8602 | 0.02823 | 0.000222 | 44.47572 |
| 54 | rs17168486 | 0.028 | 0.176 | 4.17E-36 | 0.5088 | 0.693 | 0.5616 | 0.000227 | 45.63089 |
| 55 | rs11708067 | -0.0281 | 0.177 | 1.63E-43 | 0.2641 | 0.2775 | 0.04824 | 0.00023 | 46.16256 |
| 56 | rs7163757 | -0.0217 | 0.433 | 2.64E-36 | 0.8846 | 0.8425 | 0.630901 | 0.000231 | 46.39755 |
| 57 | rs1820176 | -0.0247 | 0.296 | 1.91E-34 | 0.01085 | 0.02654 | 0.2831 | 0.000254 | 51.02382 |
| 58 | rs10838524 | -0.0238 | 0.52 | 1.56E-40 | 0.9383 | 0.8668 | 0.9313 | 0.000283 | 56.74473 |
| 59 | rs7903146 | 0.0259 | 0.307 | 1.99E-35 | 0.2075 | 0.645 | 0.8534 | 0.000285 | 57.27952 |
| 60 | rs9650069 | -0.0286 | 0.284 | 8.31E-58 | 0.008415 | 0.1095 | 0.02965 | 0.000333 | 66.75936 |
| 61 | rs10487796 | -0.0261 | 0.484 | 4.62E-52 | 0.4077 | 0.4854 | 0.7472 | 0.00034 | 68.28544 |
| 62 | rs1260326 | 0.0282 | 0.587 | 4.48E-65 | 0.1588 | 0.3986 | 0.5145 | 0.000386 | 77.38523 |
| 63 | rs878521 | 0.0549 | 0.249 | 2.65E-174 | 0.4069 | 0.436 | 0.6065 | 0.001127 | 226.4008 |
| 64 | rs537183 | 0.0663 | 0.64 | 1.00E-200 | 0.1948 | 0.4189 | 0.670101 | 0.002026 | 407.1874 |
| 65 | rs10830963 | 0.0772 | 0.286 | 1.00E-200 | 0.4641 | 0.2684 | 0.2742 | 0.002434 | 489.5099 |
| Confounding | |  |  |  |  |  |  |  |  |
| 1 | rs10830963 | Obesity related traits | | | | | | | |
| 2 | rs174583 | Lung cancer | | | | | | | |
| 3 | rs507666 | High grade serous ovarian cancer | | | | | | | |
| 4 | rs507666 | Serous invasive ovarian cancer | | | | | | | |

**Supplementary Table 4:** The instrumental variables of MR analysis between fasting insulin and three outcomes (venous thromboembolism, deep vein thrombosis and pulmonary embolism).

|  | **SNP** | **beta.FI** | **eaf.FI** | **pval.FI** | **pval.VTE** | **pval.DVT** | **pval.PE** | **R2** | **F** |
| --- | --- | --- | --- | --- | --- | --- | --- | --- | --- |
| 1 | rs10050393 | -0.009 | 0.46 | 4.84E-08 | 0.2408 | 0.8039 | 0.1119 | 4.02E-05 | 6.077048 |
| 2 | rs17331151 | -0.0162 | 0.106 | 1.52E-08 | 0.5695 | 0.4416 | 0.04742 | 4.97E-05 | 7.51162 |
| 3 | rs11708067 | 0.0135 | 0.177 | 1.30E-09 | 0.2641 | 0.2775 | 0.04824 | 5.31E-05 | 8.018669 |
| 4 | rs2845885 | -0.0204 | 0.931 | 1.18E-08 | 0.1892 | 0.4431 | 0.098261 | 5.35E-05 | 8.074598 |
| 5 | rs972283 | 0.0105 | 0.544 | 1.09E-08 | 0.003109 | 0.01174 | 0.008498 | 5.47E-05 | 8.260468 |
| 6 | rs2108349 | -0.0115 | 0.686 | 1.13E-08 | 0.211 | 0.000106 | 0.7259 | 5.70E-05 | 8.604245 |
| 7 | rs7903146 | -0.0116 | 0.307 | 1.24E-09 | 0.2075 | 0.645 | 0.8534 | 5.73E-05 | 8.646713 |
| 8 | rs6905288 | 0.0112 | 0.602 | 7.75E-09 | 0.6122 | 0.4497 | 0.4746 | 6.01E-05 | 9.077793 |
| 9 | rs1351394 | 0.0111 | 0.529 | 2.71E-09 | 0.8351 | 0.6003 | 0.5306 | 6.14E-05 | 9.272307 |
| 10 | rs13258890 | -0.0128 | 0.252 | 2.77E-08 | 0.4987 | 0.9537 | 0.0746 | 6.18E-05 | 9.327977 |
| 11 | rs1206760 | -0.0112 | 0.522 | 8.82E-10 | 0.3039 | 0.3746 | 0.4059 | 6.26E-05 | 9.453665 |
| 12 | rs11727676 | 0.0203 | 0.084 | 2.90E-08 | 0.1513 | 0.2596 | 0.096261 | 6.34E-05 | 9.577077 |
| 13 | rs731839 | -0.0121 | 0.658 | 3.86E-11 | 0.083389 | 0.003401 | 0.7865 | 6.59E-05 | 9.951532 |
| 14 | rs5017305 | -0.0137 | 0.764 | 1.07E-08 | 0.05494 | 0.079239 | 0.3665 | 6.77E-05 | 10.2215 |
| 15 | rs75179845 | 0.0216 | 0.079 | 6.05E-11 | 1.00E-13 | 1.44E-11 | 9.69E-07 | 6.79E-05 | 10.25327 |
| 16 | rs73013411 | -0.018 | 0.12 | 2.08E-08 | 0.4326 | 0.9152 | 0.6109 | 6.84E-05 | 10.33421 |
| 17 | rs3775380 | 0.0119 | 0.5 | 1.48E-11 | 0.248 | 0.03274 | 0.8697 | 7.08E-05 | 10.69309 |
| 18 | rs6855363 | -0.0125 | 0.347 | 4.04E-08 | 0.4878 | 0.1468 | 0.9208 | 7.08E-05 | 10.6938 |
| 19 | rs7133378 | -0.0127 | 0.339 | 6.00E-11 | 0.01333 | 0.4116 | 0.004075 | 7.23E-05 | 10.91638 |
| 20 | rs62271373 | 0.0256 | 0.059 | 1.60E-08 | 0.4138 | 0.5046 | 0.3213 | 7.28E-05 | 10.98985 |
| 21 | rs9884482 | 0.0125 | 0.392 | 2.88E-11 | 0.1595 | 0.4832 | 0.2795 | 7.45E-05 | 11.24814 |
| 22 | rs6487237 | 0.0154 | 0.79 | 4.68E-09 | 0.8355 | 0.8513 | 0.9394 | 7.87E-05 | 11.88394 |
| 23 | rs10865959 | 0.0138 | 0.3 | 1.99E-08 | 0.085749 | 0.6903 | 0.2385 | 8.00E-05 | 12.07955 |
| 24 | rs118164457 | 0.0345 | 0.037 | 3.86E-10 | 0.8654 | 0.3831 | 0.5944 | 8.48E-05 | 12.80978 |
| 25 | rs860598 | 0.0177 | 0.824 | 6.88E-12 | 0.558 | 0.5574 | 0.386 | 9.09E-05 | 13.72349 |
| 26 | rs12454712 | -0.0142 | 0.398 | 1.78E-09 | 0.8939 | 0.557799 | 0.9374 | 9.66E-05 | 14.59274 |
| 27 | rs17036126 | 0.0209 | 0.129 | 1.28E-10 | 0.2351 | 0.4804 | 0.4465 | 9.82E-05 | 14.82455 |
| 28 | rs1474696 | 0.0147 | 0.476 | 3.02E-16 | 0.9862 | 0.3702 | 0.573 | 0.000108 | 16.28015 |
| 29 | rs4865796 | 0.0165 | 0.707 | 7.33E-17 | 0.6854 | 0.7927 | 0.5938 | 0.000113 | 17.03501 |
| 30 | rs116141873 | 0.0428 | 0.032 | 1.42E-11 | 0.4907 | 0.6699 | 0.4058 | 0.000113 | 17.1396 |
| 31 | rs2780215 | -0.0392 | 0.042 | 1.06E-09 | 0.537499 | 0.892 | 0.4094 | 0.000124 | 18.6758 |
| 32 | rs459193 | 0.0181 | 0.715 | 1.12E-18 | 0.02538 | 0.02448 | 0.02708 | 0.000134 | 20.1653 |
| 33 | rs35000407 | -0.0258 | 0.117 | 1.50E-21 | 0.5031 | 0.3819 | 0.686901 | 0.000138 | 20.77229 |
| 34 | rs6674544 | 0.0177 | 0.574 | 6.97E-21 | 0.3431 | 0.4172 | 0.8095 | 0.000153 | 23.14052 |
| 35 | rs13389219 | -0.0199 | 0.409 | 5.84E-28 | 0.142 | 0.1833 | 0.672 | 0.000191 | 28.91603 |
| 36 | rs7012814 | -0.0219 | 0.471 | 8.34E-30 | 0.1454 | 0.05649 | 0.05684 | 0.000239 | 36.1 |
| 37 | rs1260326 | 0.0231 | 0.587 | 8.42E-38 | 0.1588 | 0.3986 | 0.5145 | 0.000259 | 39.08077 |
| 38 | rs2943646 | 0.025 | 0.623 | 8.47E-39 | 0.9922 | 0.4126 | 0.3099 | 0.000294 | 44.34815 |
| Confounding | |  |  |  |  |  |  |  |  |
| 1 | rs35000407 | Malignant neoplasm of other and unspecified parts of tongue | | | | | | | |

**Supplementary Table 5:** The instrumental variables of MR analysis between glycated hemoglobin and three outcomes (venous thromboembolism, deep vein thrombosis and pulmonary embolism).

|  | **SNP** | **beta.GH** | **eaf.GH** | **pval.GH** | **pval.VTE** | **pval.DVT** | **pval.PE** | **R2** | **F** |
| --- | --- | --- | --- | --- | --- | --- | --- | --- | --- |
| 1 | rs7127313 | 0.0066 | 0.336 | 4.85E-08 | 0.1039 | 0.01108 | 0.3114 | 1.94E-05 | 2.853458 |
| 2 | rs608793 | 0.0065 | 0.479 | 4.54E-08 | 0.2327 | 0.1851 | 0.7279 | 2.11E-05 | 3.095829 |
| 3 | rs79403657 | 0.009 | 0.177 | 2.03E-08 | 0.1824 | 0.8599 | 0.1097 | 2.36E-05 | 3.464466 |
| 4 | rs2001945 | 0.0069 | 0.53 | 6.18E-10 | 0.1307 | 0.02679 | 0.1114 | 2.37E-05 | 3.482171 |
| 5 | rs1535464 | -0.0086 | 0.212 | 1.11E-08 | 0.6283 | 0.4978 | 0.2926 | 2.47E-05 | 3.627752 |
| 6 | rs4727979 | -0.0121 | 0.094 | 4.61E-08 | 0.522001 | 0.358 | 0.137 | 2.49E-05 | 3.661049 |
| 7 | rs8138197 | -0.0073 | 0.488 | 3.54E-08 | 0.6996 | 0.27 | 0.693201 | 2.66E-05 | 3.909444 |
| 8 | rs737092 | 0.0073 | 0.499 | 7.57E-09 | 0.2996 | 0.7861 | 0.2124 | 2.66E-05 | 3.911681 |
| 9 | rs13234131 | 0.0113 | 0.124 | 2.06E-09 | 0.1846 | 0.9234 | 0.128 | 2.77E-05 | 4.072514 |
| 10 | rs7198799 | 0.0083 | 0.281 | 4.76E-09 | 0.4214 | 0.6271 | 0.1655 | 2.78E-05 | 4.086687 |
| 11 | rs10946402 | 0.0101 | 0.169 | 1.12E-10 | 0.04038 | 0.3767 | 0.04144 | 2.87E-05 | 4.206402 |
| 12 | rs360147 | -0.0086 | 0.264 | 2.08E-09 | 0.713899 | 0.9337 | 0.2794 | 2.87E-05 | 4.219481 |
| 13 | rs7547793 | 0.0118 | 0.88 | 6.61E-09 | 0.519501 | 0.858 | 0.1891 | 2.94E-05 | 4.317264 |
| 14 | rs1278769 | 0.0091 | 0.769 | 5.51E-12 | 0.778099 | 0.3769 | 0.2838 | 2.94E-05 | 4.319185 |
| 15 | rs3829109 | -0.0086 | 0.276 | 2.68E-08 | 0.5756 | 0.3599 | 0.1582 | 2.96E-05 | 4.339356 |
| 16 | rs11643024 | -0.0084 | 0.697 | 7.97E-10 | 0.1404 | 0.06491 | 0.7582 | 2.98E-05 | 4.37537 |
| 17 | rs151165 | 0.0079 | 0.397 | 2.04E-09 | 0.2833 | 0.835 | 0.3803 | 2.99E-05 | 4.38675 |
| 18 | rs11039154 | -0.0087 | 0.277 | 3.10E-09 | 0.1661 | 0.1242 | 0.3568 | 3.03E-05 | 4.450795 |
| 19 | rs13419763 | 0.008 | 0.588 | 5.48E-09 | 0.39 | 0.7545 | 0.2702 | 3.10E-05 | 4.552352 |
| 20 | rs1175549 | -0.0098 | 0.214 | 7.13E-13 | 0.2681 | 0.5736 | 0.02462 | 3.23E-05 | 4.743189 |
| 21 | rs3778321 | -0.0106 | 0.176 | 4.18E-11 | 0.3502 | 0.416 | 0.727601 | 3.26E-05 | 4.784468 |
| 22 | rs204995 | 0.0098 | 0.219 | 1.93E-09 | 0.3195 | 0.698101 | 0.6675 | 3.29E-05 | 4.823135 |
| 23 | rs10151436 | -0.013 | 0.11 | 3.85E-11 | 0.05696 | 0.09758 | 0.1953 | 3.31E-05 | 4.857934 |
| 24 | rs6877043 | -0.0085 | 0.362 | 1.99E-10 | 0.5539 | 0.6112 | 0.9083 | 3.34E-05 | 4.899474 |
| 25 | rs340882 | 0.0084 | 0.58 | 1.48E-10 | 0.01203 | 0.000715 | 0.6574 | 3.44E-05 | 5.04683 |
| 26 | rs2375278 | -0.0112 | 0.824 | 1.05E-11 | 0.136 | 0.447 | 0.3798 | 3.64E-05 | 5.341455 |
| 27 | rs267738 | -0.0109 | 0.203 | 1.14E-11 | 0.01665 | 0.05133 | 0.074199 | 3.84E-05 | 5.644064 |
| 28 | rs651007 | 0.0108 | 0.215 | 3.28E-15 | 3.06E-46 | 1.88E-32 | 4.08E-17 | 3.94E-05 | 5.78017 |
| 29 | rs12491937 | -0.009 | 0.445 | 1.42E-13 | 0.5986 | 0.3433 | 0.608199 | 4.00E-05 | 5.873856 |
| 30 | rs10774624 | 0.0093 | 0.525 | 4.17E-14 | 0.01008 | 0.01259 | 0.8352 | 4.31E-05 | 6.332941 |
| 31 | rs1367173 | -0.0152 | 0.106 | 1.66E-14 | 0.9379 | 0.4763 | 0.122 | 4.38E-05 | 6.428616 |
| 32 | rs11257655 | 0.011 | 0.241 | 1.91E-13 | 0.01732 | 0.01903 | 0.4437 | 4.43E-05 | 6.498772 |
| 33 | rs452306 | -0.0098 | 0.627 | 5.51E-13 | 0.02559 | 0.07374 | 0.04615 | 4.49E-05 | 6.595017 |
| 34 | rs10811661 | -0.0128 | 0.165 | 1.74E-14 | 0.881 | 0.9269 | 0.738999 | 4.51E-05 | 6.627929 |
| 35 | rs174559 | -0.0106 | 0.285 | 3.31E-13 | 0.02779 | 0.071469 | 0.09808 | 4.58E-05 | 6.722803 |
| 36 | rs6804915 | -0.0108 | 0.288 | 2.76E-16 | 0.9219 | 0.2895 | 0.7055 | 4.78E-05 | 7.022773 |
| 37 | rs2015803 | 0.0112 | 0.265 | 2.28E-12 | 0.3812 | 0.8654 | 0.5525 | 4.89E-05 | 7.17395 |
| 38 | rs11720108 | -0.0129 | 0.182 | 2.95E-18 | 0.4012 | 0.3034 | 0.111 | 4.95E-05 | 7.274339 |
| 39 | rs9914988 | 0.0125 | 0.802 | 4.66E-17 | 0.6295 | 0.8936 | 0.6699 | 4.96E-05 | 7.285327 |
| 40 | rs7042939 | -0.0102 | 0.582 | 1.50E-15 | 0.4782 | 0.729699 | 0.4053 | 5.06E-05 | 7.431722 |
| 41 | rs7861647 | 0.0128 | 0.193 | 4.50E-14 | 0.5297 | 0.2861 | 0.0259 | 5.10E-05 | 7.492743 |
| 42 | rs10169706 | 0.026 | 0.04 | 1.48E-08 | 0.9572 | 0.2112 | 0.2451 | 5.19E-05 | 7.62199 |
| 43 | rs11248914 | -0.0114 | 0.302 | 1.42E-14 | 0.5823 | 0.1454 | 0.3948 | 5.48E-05 | 8.043846 |
| 44 | rs9818758 | 0.0131 | 0.204 | 1.49E-13 | 0.6485 | 0.615601 | 0.4224 | 5.57E-05 | 8.182347 |
| 45 | rs2908277 | 0.0166 | 0.117 | 1.29E-18 | 0.9283 | 0.1405 | 0.03751 | 5.69E-05 | 8.35902 |
| 46 | rs4980325 | 0.0108 | 0.532 | 4.70E-14 | 0.3216 | 0.4594 | 0.1777 | 5.81E-05 | 8.527036 |
| 47 | rs13089972 | 0.0111 | 0.584 | 1.87E-15 | 0.8328 | 0.6374 | 0.428 | 5.99E-05 | 8.789133 |
| 48 | rs10405535 | -0.0122 | 0.71 | 6.47E-14 | 0.1208 | 0.000291 | 0.952 | 6.13E-05 | 8.998508 |
| 49 | rs61750929 | -0.0284 | 0.041 | 9.49E-24 | 0.042631 | 0.1879 | 0.123 | 6.34E-05 | 9.31182 |
| 50 | rs13389076 | 0.0332 | 0.034 | 3.04E-18 | 0.6747 | 0.654699 | 0.6867 | 7.24E-05 | 10.62996 |
| 51 | rs7903146 | 0.0133 | 0.307 | 1.04E-22 | 0.2075 | 0.645 | 0.8534 | 7.53E-05 | 11.05034 |
| 52 | rs6474359 | -0.0427 | 0.022 | 1.91E-33 | 0.3255 | 0.4405 | 0.596499 | 7.85E-05 | 11.51912 |
| 53 | rs17533945 | 0.0128 | 0.418 | 1.62E-23 | 0.734401 | 0.9943 | 0.8658 | 7.97E-05 | 11.70366 |
| 54 | rs4760682 | 0.0164 | 0.817 | 3.20E-20 | 0.469 | 0.345 | 0.93 | 8.04E-05 | 11.80766 |
| 55 | rs117233107 | -0.047 | 0.02 | 8.45E-11 | 0.543599 | 0.1264 | 0.5961 | 8.66E-05 | 12.71327 |
| 56 | rs12612492 | 0.0188 | 0.148 | 1.88E-26 | 0.9796 | 0.606001 | 0.9551 | 8.91E-05 | 13.0865 |
| 57 | rs13134327 | 0.0144 | 0.331 | 2.81E-26 | 0.3367 | 0.9432 | 0.2035 | 9.18E-05 | 13.48301 |
| 58 | rs11558471 | -0.0151 | 0.293 | 3.38E-25 | 0.008948 | 0.1113 | 0.03817 | 9.45E-05 | 13.86915 |
| 59 | rs76533333 | 0.0265 | 0.087 | 2.81E-29 | 0.4928 | 0.4965 | 0.3057 | 0.000112 | 16.3794 |
| 60 | rs1800562 | -0.0383 | 0.046 | 2.33E-50 | 0.072191 | 0.04709 | 0.1646 | 0.000129 | 18.90286 |
| 61 | rs837763 | 0.0176 | 0.578 | 5.20E-38 | 0.502 | 0.01321 | 0.608001 | 0.000151 | 22.18703 |
| 62 | rs10830963 | 0.0197 | 0.286 | 1.54E-36 | 0.4641 | 0.2684 | 0.2742 | 0.000158 | 23.27198 |
| 63 | rs855791 | -0.0188 | 0.6 | 1.34E-56 | 0.771299 | 0.5133 | 0.8455 | 0.00017 | 24.9097 |
| 64 | rs857725 | 0.0208 | 0.277 | 5.43E-55 | 0.6332 | 0.8069 | 0.2632 | 0.000173 | 25.44415 |
| 65 | rs4737009 | 0.0228 | 0.262 | 8.29E-56 | 0.1729 | 0.3692 | 0.7992 | 0.000201 | 29.5177 |
| 66 | rs9376090 | -0.0247 | 0.272 | 1.90E-62 | 0.06121 | 0.01801 | 0.1947 | 0.000242 | 35.47864 |
| 67 | rs2613522 | 0.027 | 0.26 | 9.34E-39 | 0.3723 | 0.2454 | 0.4557 | 0.000281 | 41.1929 |
| 68 | rs2971670 | 0.0316 | 0.181 | 5.10E-88 | 0.3124 | 0.2466 | 0.740399 | 0.000296 | 43.47435 |
| 69 | rs560887 | 0.0307 | 0.694 | 5.55E-122 | 0.05207 | 0.1611 | 0.4569 | 0.0004 | 58.78945 |
| 70 | rs9909940 | 0.0322 | 0.323 | 1.43E-116 | 0.05644 | 0.05955 | 0.1209 | 0.000453 | 66.59901 |
| 71 | rs17476364 | -0.0858 | 0.099 | 1.00E-200 | 0.3133 | 0.01018 | 0.5595 | 0.001313 | 193.0515 |
| Confounding | |  |  |  |  |  |  |  |  |
| 1 | rs10830963 | Obesity related traits | | | | | | | |
| 2 | rs651007 | Serous invasive ovarian cancer | | | | | | | |
| 3 | rs651007 | High grade serous ovarian cancer | | | | | | | |
| 4 | rs651007 | Invasive ovarian cancer | | | | | | | |

**Supplementary Table 6:** MR analysis process of the exposure (type 2 diabetes, fasting glucose, fasting insulin, glycated hemoglobin) and outcome (venous thromboembolism, deep vein thrombosis, pulmonary embolism).

| **Exposure** | **Outcome** | **Heterogeneity Test** | | **Pleiotropy Test** | **MR-PRESSO** | | | **Leave**  **One**  **Out** |
| --- | --- | --- | --- | --- | --- | --- | --- | --- |
|  |  | **Cochran’s Q Test (*P* value)** | **Rucker’s** **Q Test (*P* value)** | **Egger Intercept**  **(*P* value)** | **Distortion Test** | **Distortion Test** | **Global Test** |  |
|  |  | **IVW** | **MR-Egger** | **MR-Egger** | **Outliers** | **Potential outliers** | ***P* value** |  |
| T2D 1 | VTE | 0.190 | 0.142 | 0.968 | 0 | 0 | 0.253 | 2 |
| T2D 2 |  | 0.403 | 0.371 | 0.454 | 0 | 0 | 0.451 | 1 |
| T2D 3 |  | 0.642 | 0.565 | 0.684 | 0 | 0 | 0.702 | 5 |
| FG 1 |  | 0.615e-3 | 0.457e-3 | 0.829 | 1 | 1 | <0.001 | 11 |
| FG 2 |  | 0.019 | 0.015 | 0.943 | 0 | 2 | 0.012 | 0 |
| FI 1 |  | 0.131 | 0.217 | 0.095 | 0 | 0 | 0.148 | 1 |
| FI 2 |  | 0.233 | 0.310 | 0.133 | 0 | 0 | 0.231 | 8 |
| FG 1 | DVT | 0.525e-3 | 0.381e-3 | 0.973 | 1 | 2 | 0.002 | 0 |
| FG 2 |  | 0.017 | 0.014 | 0.729 | 0 | 3 | 0.013 | 0 |
| FI 1 |  | 0.431 | 0.541 | 0.109 | 0 | 0 | 0.476 | 9 |
| T2D 1 | PE | 0.464 | 0.386 | 0.941 | 0 | 0 | 0.468 | 4 |
| FG 1 |  | 0.011 | 0.008 | 0.908 | 0 | 1 | 0.018 | 0 |
| FI 1 |  | 0.001 | 0.004 | 0.093 | 2 | 1 | 0.002 | 0 |
